# The Altered Proteomic Landscape in Renal Tubular Epithelial Cells under High Oxalate Stimulation

**DOI:** 10.3390/biology13100814

**Published:** 2024-10-11

**Authors:** Sen-Yuan Hong, Bao-Long Qin

**Affiliations:** Department of Urology, Tongji Hospital, Tongji Medical College, Huazhong University of Science and Technology, Wuhan 430030, China

**Keywords:** calcium oxalate stones, oxidative stress, ferroptosis, vitamin D, proteomics

## Abstract

**Simple Summary:**

This study explores how high oxalate levels, a key factor in calcium oxalate stone formation, affect renal tubular epithelial cells. Using proteomics, we identified 132 up-regulated and 136 down-regulated proteins in oxalate-treated cells. These changes were associated with critical pathological processes, including oxidative stress, apoptosis, ferroptosis, pro-inflammatory cytokines production, and vitamin D-related biomineralization, which all contribute to kidney damage and stone formation. The study also highlighted specific proteins, like SPP1, MFGE8, ANKS1A, NAP1L1, SUB1, RNPS1, and DGLUCY, which may serve as potential biomarkers or therapeutic targets. By providing novel insights into the altered proteomic landscape under high oxalate conditions, this study offers a deeper understanding of the molecular effects of high oxalate levels on rat renal tubular epithelial cells and points to new avenues for prevention and treatment strategies in calcium oxalate stone formation.

**Abstract:**

Our study aimed to apply a proteomic approach to investigate the molecular mechanisms underlying the effects of oxalate on rat renal tubular epithelial cells. NRK-52E cells were treated with or without oxalate and subjected to quantitative proteomics to identify key proteins and key pathological changes under high oxalate stimulation. A total of 268 differentially expressed proteins (DEPs) between oxalate-treated and control groups were identified, with 132 up-regulated and 136 down-regulated proteins. Functional enrichment analysis revealed that DEPs are associated with oxidative stress, apoptosis, ferroptosis, pro-inflammatory cytokines, vitamin D, and biomineralization. SPP1, MFGE8, ANKS1A, and NAP1L1 were up-regulated in the oxalate-treated cells and the hyperoxaluric stone-forming rats, while SUB1, RNPS1, and DGLUCY were down-regulated in both cases. This altered proteomic landscape sheds light on the pathological processes involved in oxalate-induced renal damage and identifies potential biomarkers and therapeutic targets to mitigate the effects of hyperoxaluria and reduce the risk of CaOx stone formation.

## 1. Introduction

Urolithiasis is a common urological condition, with calcium oxalate (CaOx) being the most prevalent type of stone [1]. The process of CaOx stone formation is multifactorial and involves urine supersaturation, crystal nucleation, growth, and retention in the urinary tract [2]. Hyperoxaluria is a significant risk factor for CaOx stone formation, which can be primary (due to a genetic defect in oxalate metabolism) or secondary (due to increased dietary oxalate intake, malabsorption syndromes, or certain metabolic disorders) [3]. Oxalate can combine with calcium in the urine to form crystals, which may eventually lead to stone formation and development. Also, hyperoxaluria can directly act on renal tubular epithelial cells (RTECs) and induce several pathological changes. Oxalate exposure can induce oxidative stress, autophagy dysfunction, and excessive apoptosis in RTECs to further mediate cellular damage [4,5,6]. The injured RTECs lose their ability to prevent crystal adhesion, allowing crystal adhesion and retention. However, the molecular mechanisms by which oxalate induces cellular damage in RTECs remain poorly understood.

Proteomics, a powerful tool for large-scale protein analysis, allows for the identification and quantification of proteins that are differentially expressed in response to specific stimuli [7]. This comprehensive analysis provides a deeper understanding of the molecular processes involved in cellular responses to pathological conditions. Several studies have been conducted to investigate the cellular response to solid CaOx crystals using the proteomics. For example, Wang et al. explored the proteomic changes in RTECs under the stimulation of CaOx crystals, highlighting the key proteins involved in cytoskeletal rearrangement and focal adhesion that could help explain how crystals adhere to RTECs, a critical step in stone formation [8]. However, few studies examined the effects of soluble oxalate on RTECs in terms of proteomic alterations and associated functions and pathways. Our study innovatively employed a proteomic approach to explore how oxalate influences protein expression and how these changes may contribute to the pathogenesis of CaOx stones.

## 2. Materials and Methods

### 2.1. Cell Culture and Treatment

The NRK-52E cell line was purchased from the Type Culture Collection of the Chinese Academy of Sciences (Shanghai, China) and maintained in a DMEM medium (Gibco, Waltham, MA, USA), supplemented with 10% fetal bovine serum (Gibco, Waltham, MA, USA), at 37 °C in a humidified incubator with 5% CO_2_. The control group (*n* = 3) consisted of untreated NRK-52E cells, while the experimental group (*n* = 3) involved treatment with 1 mM oxalate (Sigma (Sofia, Bulgaria), 223433) for 24 h.

### 2.2. Protein Extraction

NRK-52E cells were rinsed with PBS three times and then harvested using cell scrapers. After collection, cells were resuspended in 1 mL of PBS and centrifuged at 1000× *g* for 5 min at 4 °C. The supernatant was discarded, and the remaining cell pellets were stored at −80 °C for protein extraction. For the extraction, the samples were mixed with 8 M urea in 100 mM Tris-Cl and subjected to water bath sonication. Following sonication, the samples were centrifuged, and the supernatant was collected for a reduction reaction (10 mM DTT at 37 °C for 1 h), followed by alkylation (40 mM iodoacetamide at room temperature for 30 min in the dark). Protein concentrations were determined using the Bradford method. The total protein quantities for each sample were as follows: Ctrl-1: 1545.6 µg, Ctrl-2: 1259.0 µg, Ctrl-3: 1404.9 µg, Ox-1: 1347.6 µg, Ox-2: 1371.0 µg, and Ox-3: 1566.4 µg. For proteomic analysis, 1000 µg of protein per sample was used. Urea concentration was diluted below 2 M using 100 mM Tris-HCl (pH 8.0), and trypsin was added at a 1:50 enzyme-to-protein ratio for overnight digestion at 37 °C. The following day, digestion was stopped by adjusting the pH to 6.0 using trifluoroacetic acid (TFA). The samples were then centrifuged at 12,000× *g* for 15 min, and the supernatant was purified using a Sep-Pak C18 desalting column. The eluted peptides were vacuum-dried and stored at −20 °C for future use.

### 2.3. Liquid Chromatography with Tandem Mass Spectrometry (LC-MS/MS) Analysis in Data Independent Acquisition (DIA) Mode

Protein detection and quantitative analysis service were provided by Bioyi Biotechnology Co., Ltd. (Wuhan, China). LC-MS/MS analysis was performed using an Orbitrap Exploris 480 mass spectrometer (Thermo Fisher Scientific, Waltham, MA, USA) paired with an Easy-nLC 1200 system (Thermo Fisher Scientific, Waltham, MA, USA). Peptides were introduced via an autosampler and separated on a C18 analytical column. The separation gradient was established with mobile phase A (0.1% formic acid) and mobile phase B (80% acetonitrile with 0.1% formic acid), at a flow rate of 300 nL/min. For the Data-Independent Acquisition (DIA) mode, each scan cycle included one full-scan mass spectrum (resolution: 60,000, AGC target: 3e6, injection time: 30 ms, scan range: 350–1250 *m*/*z*), followed by 40 variable MS/MS events (resolution: 30,000, AGC target: 1000%, injection time: 50 ms). FAIMS CV voltage was set to −45, and collision energy for HCD was set to 30.

### 2.4. Database Search and Quantitative Data Analysis

Raw mass spectrometry data were processed using DIA Neural Network (DIA-NN) software (version 1.7.15) in a library-free approach. The rat protein sequence database from SwissProt was used for library prediction based on deep learning algorithms. MBR function was employed to create a spectral library from DIA data and then to reanalyze using this library. Proteins were considered identified if they contained at least one unique peptide, and proteins with at least two peptides were included in the quantitative analysis. The false discovery rate (FDR) threshold for identified proteins was set at <1.0%. The quantification information from DIA-NN output files was used for further analysis. After normalizing the data to the total peak intensity, multivariate statistical analysis was conducted using SIMCA-P software (version 13.0). The data were preliminarily analyzed by principal component analysis (PCA) to understand the overall differences between the two groups and the degree of variability between samples [9]. PCA was performed with the linear algebra method, in which dimension reduction and principal component extraction of quantification values for all proteins were carried out. Differentially expressed proteins (DEPs) were identified by calculating the fold change (FC) in protein levels between groups. The threshold for up-regulated DEPs was set at FC ≥ 1.5 with a *p*-value ≤ 0.05 (paired two-sided Student’s t-test), and for down-regulated DEPs, the threshold was FC ≤ 0.67 with a *p*-value ≤ 0.05 [10,11]. Visualizations, including volcano plots and heatmaps, were created using the “ggplot2” and “pheatmap” packages in R (version 4.3.1).

### 2.5. Functional Enrichment Analysis

Gene Ontology (GO) enrichment analysis was performed to identify the biological functions of the DEPs. DEPs were mapped to various GO terms (http://www.geneontology.org/, accessed on 10 August 2024), and the number of associated proteins for each term was calculated. A hypergeometric test was applied to identify GO terms that were significantly enriched compared to the overall proteome. DEPs were also mapped to the Kyoto Encyclopedia of Genes and Genomes (KEGG) database (https://www.kegg.jp/kegg/pathway.html, accessed on 10 August 2024) to explore relevant pathways. Additionally, Gene Set Enrichment Analysis (GSEA) was performed using the “clusterProfiler” R package (version 4.3.1), which allows for the detection of subtle expression changes in protein sets, potentially yielding more robust results.

### 2.6. Construction of Protein–Protein Interaction (PPI) Network

By querying the STRING protein interaction database (http://string-db.org/, accessed on 10 August 2024), we constructed the PPI network of DEPs represented by nodes and edges. This approach allows us to extract valuable information on protein interactions at a systems level, providing comprehensive insights that are difficult to obtain from individual proteins alone. We considered an interaction relationship to exist when the combined score was greater than 400. Additionally, the top 30 genes with the highest number of adjacent nodes were visualized.

### 2.7. Real-Time Quantitative PCR (RT-qPCR)

NRK-52E cells exposed to 1 mM oxalate for 24 h were used for RT-qPCR validation. RNA was extracted using TRIzol reagent (Invitrogen, Waltham, MA, USA), and complementary DNA (cDNA) was synthesized using the Yeasen cDNA synthesis kit (11141ES, Shanghai, China). RT-qPCR was then performed using SYBR Green Master Mix (Yeasen, Shanghai, 11202ES, China) on the QuantStudio 6 Flex system. The relative gene expression was calculated using the 2^−ΔΔCt^ method, normalized against β-actin. The primer sequences were as follows: β-actin forward, CACGATGGAGGGGCCGGACTCATC; reverse, TAAAGACCTCTATGCCAACACAGT; ANKS1A forward, AGAAGACGGGTCCAGAAGCAGAG; reverse, GGAGCAGGTCAGGAGGTCAGAG.

### 2.8. Western Blot (WB)

NRK-52E cells treated with 1 mM oxalate for 24 h were also subjected to WB. Proteins were extracted using RIPA buffer (Boster, AR0102, Wuhan, China) containing 1% PMSF (Boster, AR1192, Wuhan, China). Protein concentration was determined using a BCA assay kit (Boster, AR1189, Wuhan, China). Equal protein amounts were separated on 10% SDS-PAGE gels and transferred to PVDF membranes (Millipore, Burlington, MA, USA). Membranes were blocked with 5% bovine serum albumin for 2 h at room temperature and incubated overnight at 4 °C with primary antibodies, including SERPINE1 (Affinity, DF13553, Changzhou, China), PTGS2 (Proteintech, 12375-1-AP, Wuhan, China), SPP1 (Proteintech, 22952-1-AP, Wuhan, China), F3 (ABclonal, A4395, Wuhan, China), RIPK3 (ABclonal, A5431, Wuhan, China), MFGE8 (ABclonal, A26854, Wuhan, China), NAP1L1 (ABclonal, A6174, Wuhan, China), SUB1 (ABclonal, A7070, Wuhan, China), RNPS1 (Cusabio, CSB-PA019907GA01HU, Wuhan, China), DGLUCY (ABclonal, A23871, Wuhan, China), and β-actin (Proteintech, 66009-1-Ig, Wuhan, China). The membranes were then incubated with horseradish peroxidase-conjugated secondary antibodies for 2 h at room temperature. Protein bands were visualized using an ECL kit (Yeasen, 36208ES, Shanghai, China), and relative protein expression was quantified using ImageJ software (version 1.8.0), normalized to β-actin.

### 2.9. Validation of DEPs in the Public Proteome Dataset

The public proteome dataset from Zhu et al. was used to validate the DEPs [12]. This dataset includes kidney samples from three normal adult Sprague-Dawley male rats and three adult male rats with hyperoxaluria-induced urolithiasis. The thresholds for identifying dysregulated proteins in their study were set at FC ≥ 1.5 with a *p*-value ≤ 0.05 for up-regulation and FC ≤ 0.67 with a *p*-value ≤ 0.05 for down-regulation. We took the intersection of the up-regulated DEPs of the oxalate-treated NRK-52E cells and the up-regulated DEPs of the stone-forming rats. Similarly, we also intersected the down-regulated genes from both conditions.

## 3. Results

### 3.1. Overview of Protein Identification

**Figure 1:**
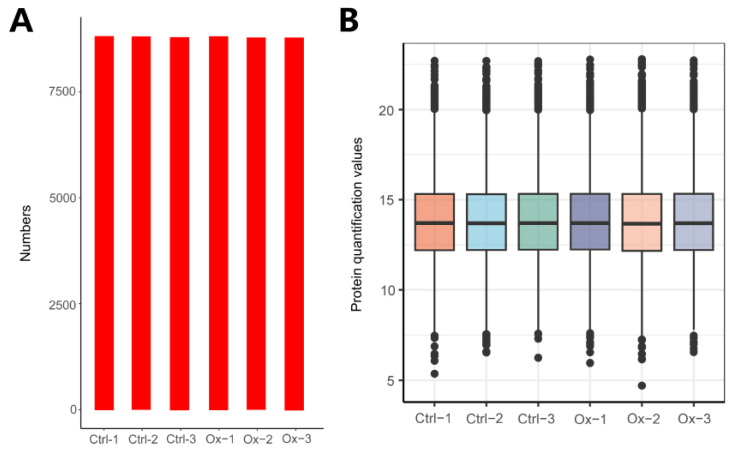
Overview of protein identification. (**A**) The total number of proteins identified in each sample: Ctrl-1: 8826, Ctrl-2: 8809, Ctrl-3: 8803, Ox-1: 8822, Ox-2: 8786, and Ox-3: 8800. (**B**) The boxplot of protein quantitative values of each sample. The X-axis represents different samples, and the Y-axis indicates Log2-transformed relative protein quantitation values. Each dot represents one protein identified by DIA proteomics. The figure measures the protein expression level of each sample from the overall dispersion of the quantification values.

A total of 8973 proteins were identified in our study, and the number of proteins identified in each sample was shown in Figure 1A. The distribution of protein quantification values was presented in Figure 1B, revealing that most samples exhibit a similar response intensity in their protein quantification values.

### 3.2. Identification of DEPs

**Figure 2:**
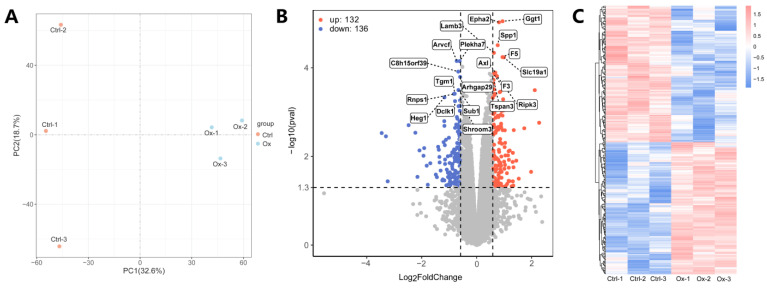
Identification of DEPs. (**A**) The PCA plot showing the distribution of the two groups. (**B**) The volcano plot of DEPs. (**C**) The heatmap of DEPs.

The PCA plot of the different samples in the control and experimental groups showed that the oxalate-treated cells exhibit distinct clustered distributions, suggesting that high oxalate stimulation induced significant cellular changes, leading to group-specific responses (Figure 2A). A total of 268 DEPs were identified, of which 132 were up-regulated and 136 were down-regulated. The list of the 268 DEPs was presented in Table S1. DEPs were presented in the volcano plot and the heatmap (Figure 2B,C). The heatmap with the detailed protein list is given in Figure S1.

### 3.3. Functional Enrichment Analysis

**Figure 3:**
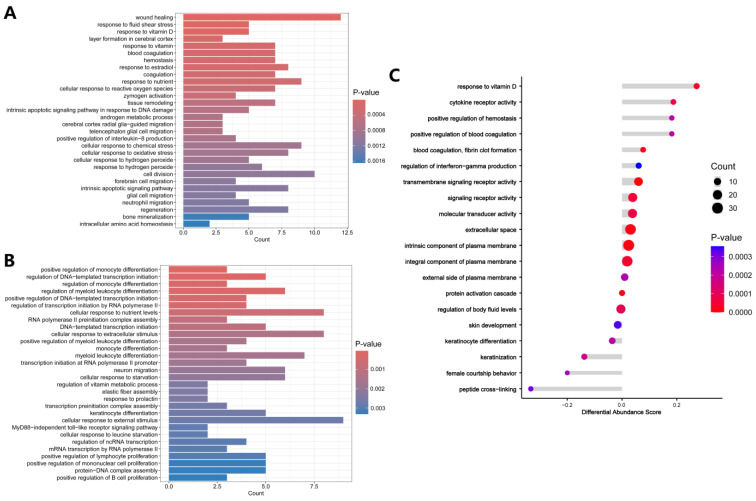
GO enrichment analysis. (**A**) GO analysis of up-regulated DEPs. (**B**) GO analysis of down-regulated DEPs. (**C**) GO analysis shown as Differential Abundance (DA) scores. A DA score greater than 0 indicates that the expression trend of all annotated proteins in the terms is up-regulated, and a DA score less than 0 indicates that the expression trend of all annotated proteins in the term is down-regulated.

The up-regulated and down-regulated DEPs were separately subjected to GO and KEGG enrichment analysis to determine functions and pathways. For GO analysis, the up-regulated DEPs were mainly enriched in response to vitamin D, cellular response to reactive oxygen species (ROS), response to oxidative stress, intrinsic apoptotic signaling pathway, positive regulation of interleukin-8 (IL-8) production, and bone mineralization. (Figure 3A). The detailed results of GO enrichment analysis of the up-regulated DEPs are presented in Table S2. The down-regulated DEPs were mainly enriched in regulation of monocyte differentiation, regulation of DNA-templated transcription initiation, regulation of myeloid leukocyte differentiation, cellular response to nutrient levels, and cellular response to extracellular stimulus (Figure 3B). The detailed results of GO enrichment analysis of the down-regulated DEPs are presented in Table S3.

GO analysis was further assessed using Differential Abundance (DA) scores. A DA score greater than 0 indicates that the expression trend of all DEPs in the term is up-regulated, while a DA score less than 0 indicates that the expression trend of all DEPs in the term is down-regulated. Figure 3C showed that DEPs associated with response to vitamin D, cytokine receptor activity, positive regulation of hemostasis, positive regulation of blood coagulation, blood coagulation, fibrin clot formation, and regulation of interferon-gamma production were up-regulated in the oxalate-treated cells.

**Figure 4:**
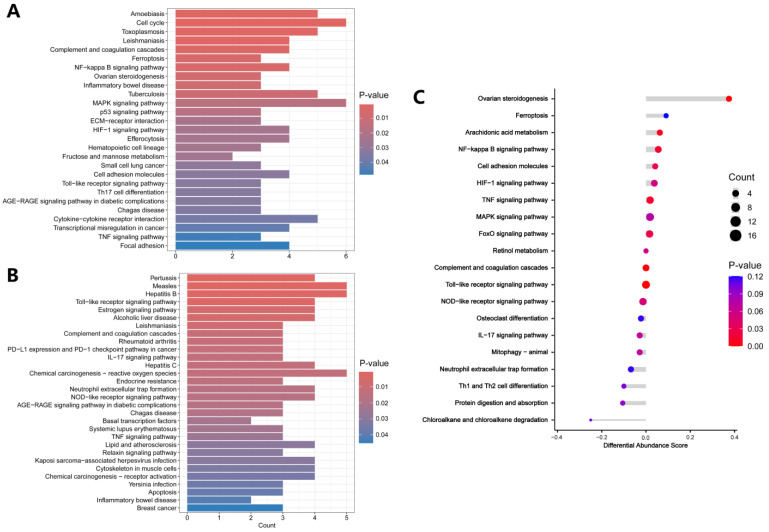
KEGG enrichment analysis. (**A**) KEGG analysis of up-regulated DEPs. (**B**) KEGG analysis of down-regulated DEPs. (**C**) KEGG analysis shown as Differential Abundance (DA) scores. A DA score greater than 0 indicates that the expression trend of all annotated proteins in the pathway is up-regulated, and a DA score less than 0 indicates that the expression trend of all annotated proteins in the pathway is down-regulated.

For KEGG analysis, the up-regulated DEPs were mainly enriched in the amoebiasis, cell cycle, toxoplasmosis, leishmaniasis, complement and coagulation cascades, and ferroptosis (Figure 4A). The detailed results of KEGG enrichment analysis of the up-regulated DEPs are presented in Table S4. The down-regulated DEPs were enriched in pertussis, measles, hepatitis B, the Toll-like receptor signaling pathway, the estrogen signaling pathway, and alcoholic liver disease (Figure 4B). The detailed results of KEGG enrichment analysis of the down-regulated DEPs are presented in Table S5. Figure 4C showed that DEPs associated with ovarian steroidogenesis, arachidonic acid metabolism, NF-κB signaling pathway, and cell adhesion molecules were up-regulated in the oxalate-treated cells.

**Figure 5:**
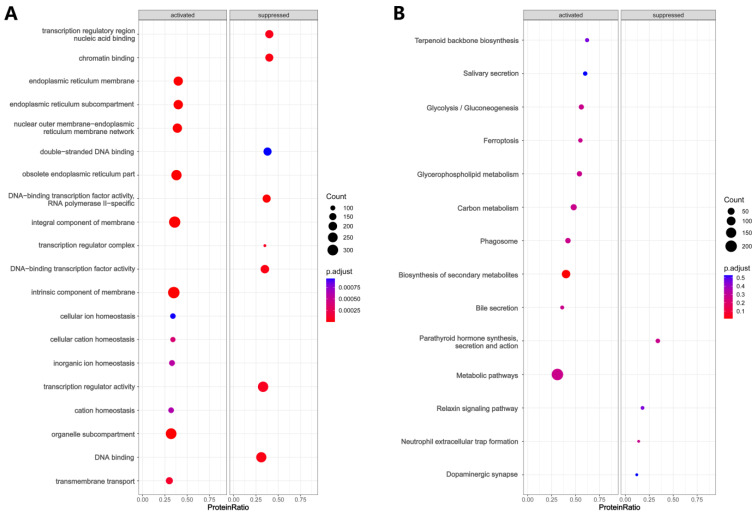
GSEA enrichment analysis. (**A**) The term “activated” refers to the up-regulation or enhancement of specific GO terms in the oxalate-treated cells compared to untreated controls, while “suppressed” denotes the down-regulation or inhibition of specific GO terms in the oxalate-treated cells compared to untreated controls. (**B**) The term “activated” refers to the up-regulation or enhancement of specific KEGG pathways in the oxalate-treated cells compared to untreated controls, while “suppressed” denotes the down-regulation or inhibition of specific KEGG pathways in the oxalate-treated cells compared to untreated controls.

The entire protein expression profile of the two groups was subjected to GSEA to explore the activation or suppression of different GO terms and KEGG pathways in the two groups. For GO terms, endoplasmic reticulum-related terms, such as endoplasmic reticulum subcompartment and endoplasmic reticulum membrane, and ion-related terms, such as cellular cation homeostasis and cellular ion homeostasis were activated in the oxalate-treated cells (Figure 5A). DNA binding-related terms, such as transcription regulatory region nucleic acid binding and chromatin binding were suppressed in the oxalate-treated cells (Figure 5A). For KEGG pathways, biosynthesis of secondary metabolites, ferroptosis, bile secretion, and glycerophospholipid metabolism were activated, while parathyroid hormone synthesis, secretion and action, neutrophil extracellular trap formation, relaxin signaling pathway, and dopaminergic synapse were suppressed in oxalate-treated cells (Figure 5B).

### 3.4. Construction of PPI Network

**Figure 6:**
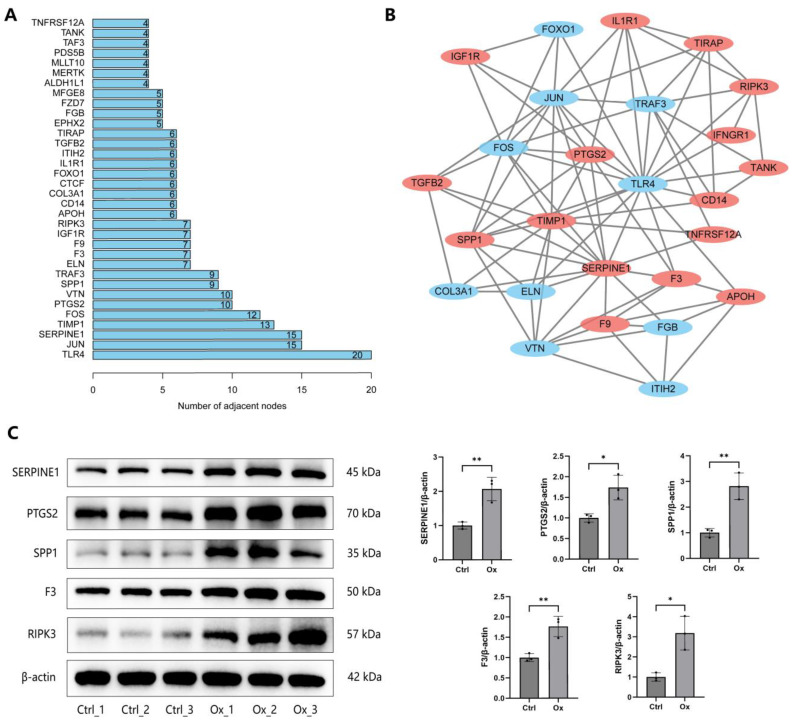
Construction of PPI network. (**A**) The top 30 DEPs with the largest number of adjacent nodes. (**B**) The most significant subnetwork identified by MCODE plugin of Cytoscape. Up-regulated and down-regulated DEPs were labeled with red and blue, respectively. (**C**) The expression of the SERPINE1, PTGS2, SPP1, F3, and RIPK3 proteins was detected by WB in NRK-52E cells after treatment with 1 mM oxalate for 24 h. * represents *p* < 0.05, ** represents *p* < 0.01.

DEPs were subjected to PPI network construction using Cytoscape to explore the relationship among them based on information in the STRING database. The specific PPI information of DEPs was presented in Table S6. The top 30 DEPs with the largest number of adjacent nodes were visualized in Figure 6A, including TLR4, JUN, SERPINE1, TIMP1, FOS, VTN, PTGS2, SPP1, TRAF3, ELN, and so on. We further used the MCODE plugin of Cytoscape to identify the most significant subnetwork in the entire PPI network, which was composed of 16 up-regulated DEPs (labeled with red) and 10 down-regulated DEPs (labeled with blue) (Figure 6B). We found that all proteins in this subnetwork are in the top 30 DEPs, except IFNGR1. Moreover, proteins in this subnetwork were encompassed in oxidative stress, apoptosis, pro-inflammatory cytokines production, vitamin D, and bone mineralization-related GO terms identified in Figure 3. In addition, four small subnetworks were also identified (Figure S2).

We further conducted WB experiments to confirm the reliability of the results of quantitative proteomics and the functional enrichment analysis. We first extracted DEPs in “cellular response to oxidative stress” (DAXX, GGT1, GLRX2, SERPINE1, RIPK3, AXL, F3, PEX2), “intrinsic apoptotic signaling pathway” (DAXX, PTGS2, STEAP3, BRCA2, SFN, EPHA2, CDKN2d, RIPK3), “response to vitamin D” (TPCN2, PTGS2, TGFB2, CDKN2D, SPP1), “bone mineralization” (PTGS2, SNX10, GPNMB, SPP1, BMP2K), and “positive regulation of interleukin-8 production” (TIRAP, SERPINE1, F3, CD14) GO terms. Then, DEPs that were encompassed in at least 2 GO terms and belonged to the top 30 DEPs were finally selected for validation, including SERPINE1, PTGS2, SPP1, F3, and RIPK3. The five selected DEPs were significantly up-regulated in oxalate-treated cells compared to untreated controls, consistent with the proteomics and the functional enrichment analysis results (Figure 6C).

### 3.5. Validation of DEPs

**Figure 7:**
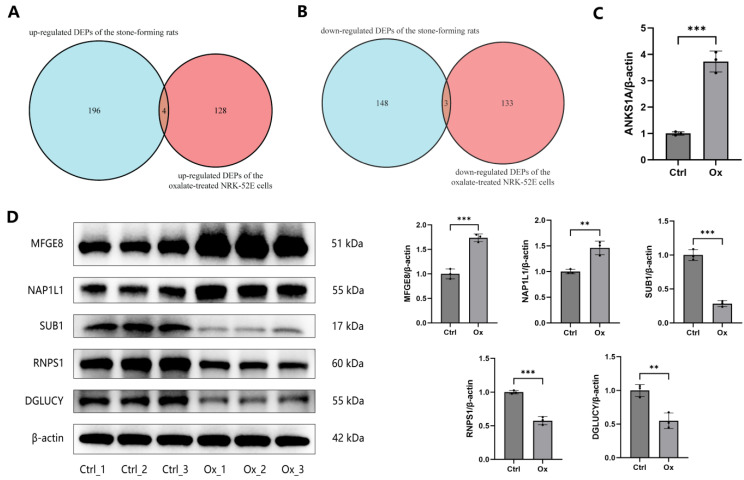
Validation of DEGs in the public proteome dataset. (**A**) The intersection of the up-regulated DEPs of the oxalate-treated NRK-52E cells and the up-regulated DEPs of the stone-forming rats. (**B**) The intersection of the down-regulated DEPs of the oxalate-treated NRK-52E cells and the down-regulated DEPs of the stone-forming rats. (**C**) The expression of the ANKS1A mRNA was detected by RT-qPCR in NRK-52E cells after treatment with 1 mM oxalate for 24 h. (**D**) The expression of the MFGE8, NAP1L1, SUB1, RNPS1, and DGLUCY proteins was detected by WB in NRK-52E cells after treatment with 1 mM oxalate for 24 h. ** represents *p* < 0.01; *** represents *p* < 0.001.

The public proteome dataset composed of three normal rats and three hyperoxaluric stone-forming rats was applied to validate DEPs. Table S7 showed the expression levels of DEPs in oxalate-treated cells from our proteomics data and hyperoxaluric stone-forming rats from the public dataset. Four common up-regulated DEPs in both the oxalate-treated cells and the hyperoxaluric stone-forming rats were screened, including SPP1, MFGE8, ANKS1A, and NAP1L1 (Figure 7A). Three common down-regulated DEPs in both datasets were also obtained, including SUB1, RNPS1, and DGLUCY (Figure 7B). The seven DEPs, except ANKS1A, were further validated by WB. ANKS1A were validated by RT-qPCR due to the lack of suitable and specific antibody. The expression of ANKS1A mRNAs was significantly up-regulated in NRK-52E cells after treatment with oxalate (Figure 7C). The expression of SPP1, MFGE8, and NAP1L1 proteins was significantly increased, while the expression of SUB1, RNPS1, and DGLUCY proteins was significantly decreased in oxalate-treated cells compared to untreated controls, suggesting they might play pathogenic and protective roles in oxalate-induced renal damage and CaOx stone disease (Figure 6C and Figure 7D).

## 4. Discussion

RTECs play a crucial role in maintaining kidney function by participating in processes such as ion transport, reabsorption, and waste excretion. However, under certain pathological conditions, these cells are vulnerable to injury and dysfunction, leading to various renal diseases. One such condition is hyperoxaluria, a significant risk factor for CaOx stones. High urinary oxalate levels stress RTECs and cause dysfunction. Over time, this may ultimately lead to kidney damage, stone formation and progression [13]. However, the molecular mechanisms by which oxalate affects RTECs remain poorly understood. Through this proteomic investigation, we seek to enhance our understanding of how oxalate influences protein expression and how these changes may contribute to CaOx stones formation.

Our study revealed an altered protein expression profile in RTECs under high oxalate stimulation. We identified a total of 268 DEPs, of which 132 were up-regulated and 136 down-regulated in oxalate-treated cells compared to controls. Functional enrichment analysis indicated that pathological processes related to oxidative stress, apoptosis, ferroptosis, pro-inflammatory cytokines, vitamin D, and biomineralization were implicated in oxalate-induced renal damage. Furthermore, the PPI network also pointed to the relationship between these functional abnormalities and the identified DEPs. Notably, the expression levels of five selected DEPs (SERPINE1, PTGS2, SPP1, F3, and RIPK3) were validated to be elevated in oxalate-treated cells compared to untreated controls, highlighting their potential pathogenic roles in CaOx stones formation.

Studies showed that oxalate triggered oxidative stress by inducing ROS production, lipid peroxidation, and mitochondrial dysfunction in RTECs [14,15]. Thamilselvan et al. found that oxalate-induced activation of PKC and the subsequent regulation of NADPH oxidase-mediated oxidative stress are critical pathways involved in the pathogenesis of CaOx stones [16]. Zhu et al. found that oxalate inhibited the Nrf2 nuclear translocation in RTECs to prevent the activation of the transcription of antioxidant and cytoprotective genes, leading to an accumulation of ROS and heightened oxidative damage [5].

Oxalate-induced cell death, such as apoptosis and ferroptosis, has received increasing attention. Oxalate can disrupt protein folding in the endoplasmic reticulum, leading to an accumulation of unfolded proteins. This disturbance triggers endoplasmic reticulum stress (ERS) and activates the unfolded protein response. When the stress persists, the unfolded protein response shifts from a protective response to a pro-apoptotic response [17]. Ming et al. found that oxalate-induced apoptosis was mediated through the interaction between the ERS/ROS and NF-κB pathways [18]. Ferroptosis has recently been implicated in the pathogenesis of CaOx stones [19]. A study by He et al. was the first to demonstrate that that oxalate exposure leads to glutathione depletion and increased lipid peroxidation in RTECs, both hallmarks of ferroptosis [20]. Inhibition of ferroptosis with ferrostatin-1 reduced renal injury and CaOx crystal formation, while activation of ferroptosis with erastin exacerbated the harmful effects. Song et al. found that oxalate-induced ferroptosis was accompanied by excessive activation of autophagy, and inhibition of autophagy could reduce ferroptosis, suggesting that autophagy plays a key role in regulating ferroptosis [21]. Dong et al. found that oxalate induced GSK3β expression in RTECs. GSK3β negatively regulated Nrf2 and inhibited the Nrf2 nuclear translocation, which led to oxidative stress and ferroptosis, since Nrf2 activation enhanced the expression of GPX4, an enzyme crucial in preventing lipid peroxidation and ferroptosis [22].

Our study validated the increased expression of RIPK3, SERPINE1, and PTGS2 in oxalate-treated cells, which have been associated with oxidative stress, apoptosis, and ferroptosis. RIPK3 has the capacity to translocate to the endoplasmic reticulum, resulting in IP3R-mediated calcium overload and xanthine oxidase-dependent oxidative stress, ultimately leading to endothelial apoptosis [23]. Liu et al. demonstrated that inhibition of RIPK3 significantly reduced oxidative stress, inflammation, and apoptosis in astrocytes through activation of the AMPK pathway [24]. Zhang et al. reported that lipopolysaccharide induced apoptosis in RTECs by promoting RIPK3 expression [25]. Additionally, several studies have highlighted RIPK3-mediated ferroptosis in human diseases [26,27]. SERPINE1 is recognized as a potential diagnostic biomarker of oxidative stress in colon cancer [28]. Its activation, mediated by TGF-β1, is implicated in hemin-induced apoptosis and inflammatory injury [29]. Similarly, PTGS2 has been identified as a novel biomarker of oxidative stress in the semen of leukocytospermia patients [30]. Song et al. found that inhibition of PTGS2 alleviated depression-like behaviors by reducing oxidative stress and neuronal apoptosis in rats [31]. Furthermore, the Nrf2/GPX4/PTGS2 pathway plays a regulatory role in ferroptosis [32]. Collectively, targeting RIPK3, SERPINE1, and PTGS2 may serve as potential therapeutic strategies against oxalate-induced renal injury by inhibiting oxidative stress, apoptosis, and ferroptosis.

Up-regulated DEPs were enriched in positive regulation of IL-8 production. Studies suggest that elevated levels of IL-8 in urine and serum could be associated with active kidney stone disease, which may serve as a potential biomarker for diagnosing the severity of urolithiasis and monitoring inflammatory responses [33,34]. Thongboonkerd et al. found that exosomes derived from CaOx crystal-treated macrophages enhanced IL-8 production from RTECs, which recruits neutrophils and other immune cells to the site of crystal deposits in the kidneys, thereby exacerbating inflammation [35]. SERPINE1 can stabilize the chemoattractant form of IL-8 at the cell surface and may represent a therapeutic target for novel anti-inflammatory strategies [36]. F3, also known as tissue factor, can induce IL-8 production through interaction with a tissue factor—factor VIIa [37]. Macrophage cytokine production was also enriched. Macrophages have been reported to release pro-inflammatory cytokines and other mediators that can exacerbate inflammation and contribute to the development of CaOx stones [38]. Although macrophages can engulf and try to clear stones or their fragments, this process can sometimes lead to chronic inflammation and fibrosis if the crystals are not efficiently removed.

Up-regulated DEPs were also associated with vitamin D and biomineralization. Vitamin D is essential for the regulation of calcium metabolism and mineralization, and its influence on CaOx stones’ formation is complex. Studies have shown that vitamin D is tightly linked to several urological diseases. For example, vitamin D deficiency and insufficiency are the causes of urinary tract infections [39]. Vitamin D, specifically its active form 1,25(OH)_2_D_3_, increases intestinal calcium absorption. While this is crucial for bone health, excessive vitamin D activity can lead to hypercalciuria, a significant risk factor for CaOx stones [40]. Studies have shown that the active form 1,25(OH)_2_D_3_ is significantly elevated in kidney stone patients, particularly in those with calcium-containing stones and hypercalciuric stone formers [41]. The vitamin D receptor (VDR) is a nuclear receptor that mediates the actions of vitamin D. Jia et al. found that 1,25(OH)_2_D_3_ activated VDR to promote the expression of BMP2, Runx2 and Osterix in RTECs, mediating their transformation into the osteoblast-like phenotypes [42]. Many scholars believe that the formation of CaOx stones starts with Randall plaques (RPs), which are calcium phosphate (CaP) deposits located in the renal papillary interstitium [43]. The main components of RPs are the same as bone minerals, so their formation is considered a biomineralization process. Under the influence of hyperoxaluria or hypercalciuria, RTECs can transform into osteoblast-like phenotypes and secrete CaP-rich matrix vesicles to form CaP deposits, ultimately leading to RPs and CaOx stone formation [44]. SPP1, also known as osteopontin, is confirmed to be increased in oxalate-treated cells and plays a critical role in the regulation of biomineralization through its interactions with the extracellular matrix and associated signaling pathways. On the one hand, SPP1 binds to hydroxyapatite, the mineral component of bone, thereby stabilizing the mineralized matrix [45]. On the other hand, SPP1 regulates cellular signaling pathways that promote osteoblast-like transformation [46]. Our findings suggest that the elevated expression of SPP1 induced by hyperoxaluria may enhance biomineralization in the kidneys, ultimately contributing to CaOx stone formation.

Thus, we speculated that oxidative stress, apoptosis, ferroptosis, pro-inflammatory cytokines, vitamin D, and biomineralization might play crucial roles in oxalate-induced renal damage and CaOx stones’ formation. Combined with the public proteome dataset, SPP1, MFGE8, ANKS1A, and NAP1L1 were up-regulated in the oxalate-treated cells and the hyperoxaluric stone-forming rats, while SUB1, RNPS1, and DGLUCY were down-regulated in both cases. In addition to regulating biomineralization, SPP1 has a multitude of other roles in CaOx stone disease. Increased SPP1 expression produced by RTECs in response to oxalate or CaOx crystals could facilitate the adhesion of crystals to damaged epithelial cells, thereby promoting crystal retention and stone formation [2]. SPP1 is also a pro-inflammatory chemokine, as it recruits immune cells, such as macrophages and neutrophils, to areas of crystal deposition. This inflammation may lead to further renal damage, creating conditions that promote crystal retention and stone formation [47]. Except for SPP1, all validated DEPs have not been reported in the field of urolithiasis. MFGE8 was reported to activate the TGF-β1 pathway to drive the osteoblast-like transformation of vascular smooth muscle cells, leading to vascular calcification [48]. This actions of MFGE8 may also occur in the pathogenesis of CaOx stones. SUB1 has critical functions in maintaining genomic integrity, particularly through its role in DNA repair mechanisms and ensuring genomic stability [49]. The roles of RNPS1 in alternative splicing and nonsense-mediated mRNA decay ensure that cells can produce the correct protein isoforms and prevent the accumulation of faulty mRNAs [50]. We assumed that these up-regulated and down-regulated DEPs exert their pathogenic or protective action in CaOx stone disease.

## 5. Conclusions

**Figure 8:**
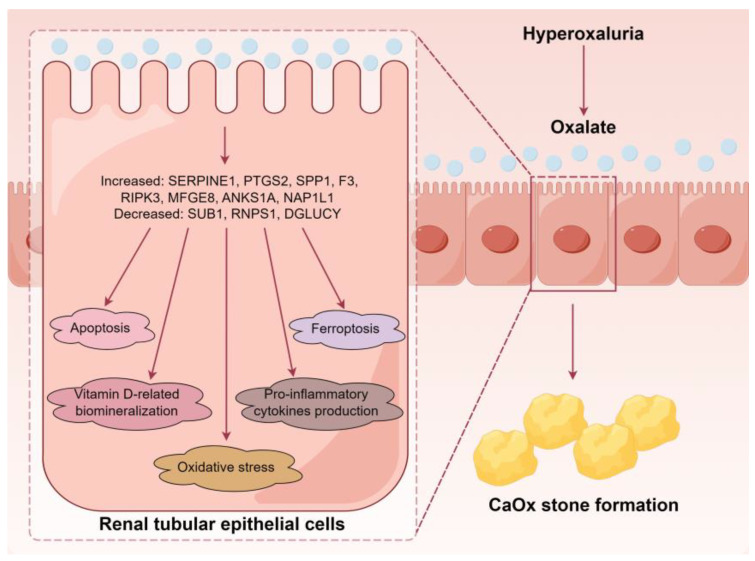
A simplified scheme illustrates the major findings of our study.

Our study provides novel insights into the altered proteomic landscape of RTECs under high oxalate stimulation, identifying key DEPs and associated pathological changes in oxalate-induced renal damage. The findings underscore the role of oxidative stress, apoptosis, ferroptosis, pro-inflammatory cytokines, vitamin D, and biomineralization in the pathogenesis of CaOx stones (Figure 8). Additionally, seven DEPs, including SPP1, MFGE8, ANKS1A, NAP1L1, SUB1, RNPS1, and DGLUCY were validated in the hyperoxaluric stone-forming rats. Ultimately, these insights could offer potential biomarkers and therapeutic targets to mitigate the effects of hyperoxaluria and reduce the risk of CaOx stone formation.

## Data Availability

Data are contained within the article.

## Supplementary Materials

The following are available online at https://www.mdpi.com/article/10.3390/biology13100814/s1, Figure S1. The heatmap with the detailed protein list. Figure S2. Four small subnetworks identified in the entire PPI network. Table S1. The list of the 268 DEPs. Table S2. The detailed results of GO enrichment analysis of the up-regulated DEPs. Table S3. The detailed results of GO enrichment analysis of the down-regulated DEPs. Table S4. The detailed results of KEGG enrichment analysis of the up-regulated DEPs. Table S5. The detailed results of KEGG enrichment analysis of the down-regulated DEPs. Table S6. The specific PPI information of DEPs. Table S7. The expression levels of DEPs in oxalate-treated cells from our proteomics data and hyperoxaluric stone forming rats from the public dataset.
