# Supplementary material for: The Altered Proteomic Landscape in Renal Tubular Epithelial Cells under High Oxalate Stimulation
Source: Biology (Basel). 2024 Oct 11;13(10):814. doi: 10.3390/biology13100814 (PMC11505525; doi:10.3390/biology13100814)
Supplement: Supplementary file 1 [file biology-13-00814-s001.zip › Table S1.pdf]

Table S1. The list of the 268 DEPs.

Protein	Fold Change	P-value
Ggt1	1.92016	8.94E-06
Epha2	1.755082	9.45E-06
Spp1	1.700031	3.1E-05
Lamb3	1.544646	4.61E-05
F5	1.916783	5.74E-05
Slc19a1	1.988778	5.81E-05
Arvcf	0.604323	7.03E-05
Plekha7	0.650976	7.08E-05
C8h15orf39	0.628729	0.000123
Axl	1.6003	0.000129
F3	1.531681	0.000139
Ripk3	1.666865	0.000154
Arhgap29	0.659065	0.000161
Tspan3	1.534508	0.000189
Dhrs9	1.530635	0.000232
Cd14	1.511262	0.000238
Dnaaf2	1.553062	0.000307
Sub1	0.630874	0.000319
Sdhaf2	4.339108	0.000321
Sfn	1.814227	0.000348
Mfge8	1.789026	0.000356
Tgm1	0.575228	0.000387
Bbs9	1.592622	0.000388
Rnps1	0.563762	0.000391
Bmp2k	1.535747	0.000399
Mthfd2	1.568978	0.000412
Heg1	0.443712	0.000466
Pex2	1.50516	0.000475
Ptgs2	1.949453	0.000516
Shroom3	0.657274	0.000546
Dclk1	0.579444	0.000568
F9	4.063229	0.000591
Lpgat1	1.568595	0.00063
Hsd17b7	1.662746	0.000682
Ttyh3	0.653595	0.000727
LOC684270	0.633805	0.000738
Hnrnpk-ps1	1.955431	0.000925
Taf4	0.653897	0.000938
Glrx2	1.879336	0.000945
Cgnl1	0.487103	0.00103
Cyb5b	1.569526	0.001041
Arnt2	1.523922	0.001111

Mier3	1.836745	0.00115
Steap3	1.918301	0.001203
Trak2	1.703802	0.001212
Serpine1	1.735992	0.001291
Selenbp1	0.607563	0.001294
Crmp1	1.616745	0.00133
Cdk5rap1	1.622673	0.001336
Tle1	0.614934	0.001415
Cd320	0.24065	0.001416
Lama3	1.51945	0.001486
Fastkd3	0.44204	0.001608
Snrnp48	0.649558	0.001632
Supt5h	4.845684	0.001744
Map1lc3b	1.850417	0.001835
Rab11fip2	0.597686	0.001912
Pigb	1.542278	0.001915
Zc3hav1l	0.620526	0.001936
Fgb	0.179766	0.001976
Eln	0.443442	0.002044
Pdp2	1.842622	0.002064
Ephx2	0.659453	0.002102
Tgfb2	1.726471	0.002157
RGD1304884	1.576858	0.002189
Rabep2	0.634228	0.002193
Mmtag2	0.580702	0.002265
Brca2	1.860282	0.002267
Akap2	0.639326	0.002285
Tram1	3.331524	0.002322
Tank	1.718373	0.002376
Timp1	1.725412	0.002489
Anapc15	2.555726	0.002552
Anxa10	1.927259	0.002558
Rad18	2.034317	0.002573
Cracdl	1.632645	0.002654
Gstm7	0.621223	0.002752
Pias1	0.641775	0.002762
Fos	0.213239	0.002926
Erg28	1.567799	0.002927
Rbpms2	1.610673	0.00295
Celf2	0.66222	0.002969
Krt2	0.091242	0.002975
Fam193b	0.63859	0.003069
Igf1r	1.524232	0.003251
Foxo1	0.649315	0.003434

Krt10	0.101457	0.003522
Gabarap	0.49671	0.003798
Ier3ip1	1.808554	0.00405
Mllt10	0.645221	0.004097
Ctcf	0.666923	0.004114
Zfp36l1	0.460735	0.004216
RGD1565222	0.434657	0.004771
Ttpa	1.807555	0.004802
Mertk	1.776821	0.004898
Anks1a	2.053842	0.004953
Uvssa	0.611312	0.004996
Ank3	0.648107	0.005124
Tmem129	1.889432	0.005191
Pfkfb4	1.552326	0.005448
Atl1	0.448305	0.005578
Zcrb1	0.378696	0.006078
Tpcn2	2.024836	0.006169
E2f3	0.611504	0.006194
Ebag9	0.643906	0.006203
Vgll4	0.613728	0.006436
Vtn	0.266254	0.006505
Aldh1l1	0.566372	0.006961
Fbxl16	0.584844	0.007013
Sclt1	0.385151	0.0071
Alas1	1.750743	0.007103
Rnf167	0.654574	0.007232
Snx10	1.751291	0.007307
Ybx3	0.236599	0.007331
Sap30	2.306087	0.007375
Tmem135	1.532279	0.007434
Pdha1l1	2.24391	0.007664
Tox4	0.54984	0.007889
Micall1	1.763505	0.007929
Tcp11l2	0.540924	0.008098
Adgrg1	2.497575	0.008322
Mfsd9	1.530668	0.008358
Dqx1	0.568755	0.008391
Szrd1	0.624128	0.008705
Taf3	0.608551	0.008736
Mast1	0.622928	0.008764
Ttc30a2	0.58293	0.009096
RT1-DMb	2.209451	0.009499
Gas2l3	2.065585	0.009522
Arhgap32	0.520726	0.009544

Krt6a	0.261674	0.009673
Tnfaip8l1	1.568938	0.009759
Ppfibp2	0.666165	0.009768
Cox6c2	0.483143	0.009953
Adat1	1.863924	0.010084
Aasdh	1.524248	0.010125
Col3a1	0.619997	0.010292
Itih2	0.39095	0.010301
Adgrl3	0.571416	0.010666
Fam83d	1.517356	0.01089
Elk3	1.658674	0.010923
Pxylp1	0.574341	0.011033
Prr15l	0.637618	0.01107
Sp2	0.602481	0.011283
Tigar	1.604685	0.011442
Pias3	0.450772	0.011795
Mia2	0.581198	0.011829
Cdkn2d	1.724812	0.011932
Psmc3	2.260189	0.011952
Zfand2a	2.722962	0.012487
Hirip3	0.665926	0.012669
Cd74	0.577212	0.01278
Slc39a14	1.500958	0.01287
Znf513	0.621718	0.013132
Nexn	1.999267	0.013144
Ttll4	1.761313	0.013148
Lrp8	2.485054	0.013327
Spsb1	0.392999	0.0137
LOC108348617	1.810439	0.013801
Prr14l	0.66184	0.013946
Adgre5	0.505871	0.014128
H2aj	0.310824	0.01429
ENSRNOG00000062700	0.543736	0.014767
Fbxw2	0.450772	0.014831
Fzd7	1.825352	0.014954
Apoh	2.047057	0.015597
Bcl7a	1.760683	0.015938
Sdc2	1.605643	0.01603
Gpnmb	1.717422	0.016161
Zscan21	0.283731	0.01621
Tent5d	1.847528	0.016287
Clic3	0.651151	0.016441
C4	0.538397	0.01664
Nfasc	2.538202	0.017643

Hddc3	2.254263	0.017979
Nap1l1	1.51436	0.018828
Ocm	2.01126	0.019039
Dmwd	0.544186	0.019064
Ssna1	1.647839	0.019434
Plekhh1	1.813702	0.019496
Dglucy	0.47062	0.019885
Taf1a	0.40294	0.020916
Srek1ip1	0.58318	0.021124
Mboat7	1.578461	0.021271
Il1r1	1.543151	0.021456
Ap3m1	1.60078	0.021924
Otud3	0.591	0.021944
Cyp4v2	1.874654	0.022257
Pds5b	3.95395	0.022331
Atp2c1	1.822013	0.022703
Etl4	0.655033	0.022811
Zfp61	0.507336	0.022927
Zfpm1	0.483368	0.023302
Tceanc	0.620691	0.023512
Tnfrsf12a	1.752949	0.023771
Dync2i2	2.146837	0.023929
H3-3b	0.583068	0.024205
--	0.520307	0.024932
LOC500028	0.566319	0.025023
Krcc1	0.357127	0.025731
Fgf13	0.501334	0.026239
Rragd	0.552125	0.026328
Jagn1	1.734187	0.026548
Thra	0.577254	0.028103
Traf3	0.635999	0.02895
Krt5	0.224775	0.029011
Olfm2	0.486017	0.029242
Zcchc9	1.51603	0.029335
Rhpn1	0.366966	0.029418
Rdh10	0.558957	0.029501
Ctso	0.494124	0.02976
Tagln3	1.780599	0.029883
Tirap	1.87113	0.030335
Pdlim2	0.621833	0.030699
Zbtb2	0.516893	0.030716
Ifi47	1.761151	0.030772
Tpp2	1.589	0.030954
Mageh1	0.50291	0.031042

Znf260	2.899252	0.031821
Ubald1	0.588212	0.031859
Rbms3	0.66115	0.032284
AC109542.1	1.51214	0.032301
Dedd	0.425766	0.033118
Mcoln1	1.610278	0.034058
Ntng2	1.703617	0.034764
Mcrip2	0.442592	0.034796
Efnb1	0.42047	0.034861
Cdc14a	2.245837	0.03562
Dusp23	0.600333	0.035736
Tpgs2	2.289164	0.036118
Ppcdc	0.579736	0.036476
Hnrnpa2b1	0.106097	0.036514
Per1	2.495888	0.036566
Rhbdl3	0.509439	0.036934
Mx1	0.517885	0.037496
Cbx7	0.521296	0.037649
Haghl	0.647034	0.038035
Ifngr1	1.674358	0.038472
Dcun1d2	1.501715	0.038706
Retsat	1.624961	0.038877
Tprn	0.596674	0.039505
Jun	0.544125	0.039783
Tead3	0.568361	0.040098
Ifrd1	1.785552	0.040477
Ttc34	1.847643	0.041423
Vtcn1	0.572381	0.041481
Sft2d3	0.518367	0.041559
Daxx	2.15975	0.041712
Phactr1	0.485974	0.043296
Eml3	0.668544	0.043361
Hps4	0.291531	0.043539
Kctd7	1.602769	0.044138
Usp2	1.745822	0.044445
Cttnbp2	0.515152	0.044469
Tlr4	0.662103	0.044906
Macrod2	0.465696	0.045012
Slc29a3	0.653259	0.04509
Ttc7b	1.674227	0.045278
St7	2.529013	0.045541
Il13ra1	2.086184	0.045991
Slc25a36l1	1.617023	0.046354
Agtpbp1	0.607488	0.046471

Gspt2	0.612975	0.046669
Cep350	0.637756	0.047179
Rbmx	2.423602	0.04718
Stkld1	1.93236	0.048299
Otulinl	1.881729	0.04841
Lrp12	1.53269	0.048594
