# Supplementary material for: The Altered Proteomic Landscape in Renal Tubular Epithelial Cells under High Oxalate Stimulation
Source: Biology (Basel). 2024 Oct 11;13(10):814. doi: 10.3390/biology13100814 (PMC11505525; doi:10.3390/biology13100814)
Supplement: Supplementary file 1 [file biology-13-00814-s001.zip › Table S2.pdf]

Table S2. The detailed results of GO enrichment analysis of the up-regulated DEPs.

Category	Description	Protein Ratio	Bg Ratio	P-value	Protein ID	Count
BP	wound healing	12/114	398/18481	6.45E-06	F9/Apoh/F5/Fzd7/Mertk/Tnfrsf12a/Serpine1/Tgfb2/Timp1/Elk3/Axl/F3	12
BP	response to fluid shear stress	5/114	48/18481	1.13E-05	Ptgs2/Serpine1/Tgfb2/Spp1/F3	5
BP	response to vitamin D	5/114	63/18481	4.32E-05	Tpcn2/Ptgs2/Tgfb2/Cdkn2d/Spp1	5
BP	layer formation in cerebral cortex	3/114	16/18481	0.000121	Adgrg1/Lrp8/Mboat7	3
BP	response to vitamin	7/114	188/18481	0.000166	Tpcn2/Ptgs2/F5/Tgfb2/Cdkn2d/Spp1/Igf1r	7
BP	blood coagulation	7/114	190/18481	0.000177	F9/Apoh/F5/Mertk/Serpine1/Axl/F3	7
BP	hemostasis	7/114	194/18481	0.000201	F9/Apoh/F5/Mertk/Serpine1/Axl/F3	7
BP	response to estradiol	8/114	259/18481	0.000204	Ptgs2/Ggt1/Brca2/Tgfb2/Hsd17b7/F3/Igf1r/Arnt2	8
BP	coagulation	7/114	198/18481	0.000228	F9/Apoh/F5/Mertk/Serpine1/Axl/F3	7
BP	response to nutrient	9/114	343/18481	0.000276	Tpcn2/Ptgs2/F5/Brca2/Ttpa/Tgfb2/Cdkn2d/Spp1/Igf1r	9
BP	cellular response to reactive oxygen species	7/114	211/18481	0.000336	Daxx/Glrx2/Serpine1/Ripk3/Axl/F3/Pex2	7
BP	zymogen activation	4/114	55/18481	0.000368	F9/Apoh/Ggt1/Serpine1	4
BP	tissue remodeling	7/114	228/18481	0.000534	Epha2/Snx10/Tgfb2/Timp1/Gpnmb/Spp1/Axl	7
BP	intrinsic apoptotic signaling pathway in response to DNA damage	5/114	111/18481	0.000623	Steap3/Brca2/Sfn/Epha2/Cdkn2d	5
BP	androgen metabolic process	3/114	28/18481	0.000669	Spp1/Hsd17b7/Dhrs9	3

BP	cerebral cortex radial glia-guided migration	3/114	28/18481	0.000669	Adgrg1/Lrp8/Mboat7	3
BP	telencephalon glial cell migration	3/114	28/18481	0.000669	Adgrg1/Lrp8/Mboat7	3
BP	positive regulation of interleukin-8 production	4/114	66/18481	0.000737	Tirap/Serpine1/F3/Cd14	4
BP	cellular response to chemical stress	9/114	399/18481	0.000821	Daxx/Ptgs2/Ggt1/Glrx2/Serpine1/Ripk3/Axl/F3/Pex2	9
BP	cellular response to oxidative stress	8/114	320/18481	0.000832	Daxx/Ggt1/Glrx2/Serpine1/Ripk3/Axl/F3/Pex2	8
BP	cellular response to hydrogen peroxide	5/114	119/18481	0.000854	Daxx/Serpine1/Ripk3/Axl/F3	5
BP	response to hydrogen peroxide	6/114	185/18481	0.001012	Daxx/Glrx2/Serpine1/Ripk3/Axl/F3	6
BP	cell division	10/114	498/18481	0.001028	Pds5b/Anapc15/Cdc14a/Daxx/Brca2/Fzd7/Sfn/Tgfb2/Ssna1/Igf1r	10
BP	forebrain cell migration	4/114	73/18481	0.001077	Adgrg1/Lrp8/Axl/Mboat7	4
BP	intrinsic apoptotic signaling pathway	8/114	335/18481	0.001116	Daxx/Ptgs2/Steap3/Brca2/Sfn/Epha2/Cdkn2d/Ripk3	8
BP	glial cell migration	4/114	74/18481	0.001133	Adgrg1/Lrp8/Tgfb2/Mboat7	4
BP	neutrophil migration	5/114	128/18481	0.001184	Tirap/Jagn1/Tgfb2/Spp1/Il1r1	5
BP	regeneration	8/114	339/18481	0.001204	Apoh/Ggt1/Fzd7/Ifrd1/Serpine1/Spp1/Axl/Igf1r	8
BP	bone mineralization	5/114	137/18481	0.001601	Ptgs2/Snx10/Gpnmb/Spp1/Bmp2k	5
BP	intracellular amino acid homeostasis	2/114	10/18481	0.001643	Kctd7/Tpp2	2
BP	amyloid-beta	3/114	39/184	0.00177	Ttpa/Ifngr1/Igf1r	3

	clearance		81	7		
BP	neuron migration	6/114	209/18481	0.001886	Adgrg1/Ntng2/Axl/Il1r1/Lrp12/Pex2	6
BP	cerebral cortex radially oriented cell migration	3/114	40/18481	0.001913	Adgrg1/Lrp8/Mboat7	3
BP	positive regulation of kinase activity	4/114	86/18481	0.001977	Mertk/Epha2/Axl/Igf1r	4
BP	iron import into cell	2/114	11/18481	0.002	Steap3/Slc39a14	2
BP	negative regulation of collateral sprouting	2/114	11/18481	0.002	Ifrd1/Spp1	2
BP	regulation of fibrinolysis	2/114	11/18481	0.002	Apoh/Serpine1	2
BP	vagina development	2/114	11/18481	0.002	Mertk/Axl	2
BP	manganese ion transmembrane transport	2/114	11/18481	0.002	Atp2c1/Slc39a14	2
BP	cellular response to aldosterone	2/114	11/18481	0.002	Serpine1/Igf1r	2
BP	response to bacterial lipopeptide	2/114	12/18481	0.002391	Tirap/Cd14	2
BP	cellular response to bacterial lipoprotein	2/114	12/18481	0.002391	Tirap/Cd14	2
BP	cellular response to bacterial lipopeptide	2/114	12/18481	0.002391	Tirap/Cd14	2
BP	pericyte cell differentiation	2/114	12/18481	0.002391	Epha2/Tgfb2	2
BP	positive regulation of transferase activity	4/114	91/18481	0.00243	Mertk/Epha2/Axl/Igf1r	4
BP	response to reactive oxygen species	7/114	301/18481	0.002645	Daxx/Glrx2/Serpine1/Ripk3/Axl/F3/Pex2	7
BP	manganese ion transport	2/114	13/18481	0.002814	Atp2c1/Slc39a14	2

BP	activation-induced cell death of T cells	2/114	13/18481	0.002814	Tgfb2/Ripk3	2
BP	response to bacterial lipoprotein	2/114	13/18481	0.002814	Tirap/Cd14	2
BP	apoptotic cell clearance	3/114	46/18481	0.002861	Mfge8/Mertk/Axl	3
BP	granulocyte migration	5/114	157/18481	0.002898	Tirap/Jagn1/Tgfb2/Spp1/Il1r1	5
BP	macrophage cytokine production	3/114	47/18481	0.003042	Tirap/Tgfb2/Axl	3
BP	regulation of macrophage cytokine production	3/114	47/18481	0.003042	Tirap/Tgfb2/Axl	3
BP	regulation of interleukin-8 production	4/114	97/18481	0.003062	Tirap/Serpine1/F3/Cd14	4
BP	interleukin-8 production	4/114	98/18481	0.003178	Tirap/Serpine1/F3/Cd14	4
BP	regulation of body fluid levels	8/114	397/18481	0.003216	F9/Apoh/F5/Map1lc3b/Mertk/Serpine1/Axl/F3	8
BP	negative regulation of macrophage cytokine production	2/114	14/18481	0.00327	Tgfb2/Axl	2
BP	positive regulation of toll-like receptor 4 signaling pathway	2/114	14/18481	0.00327	Tirap/Cd14	2
BP	response to lipoteichoic acid	2/114	14/18481	0.00327	Tirap/Cd14	2
BP	cellular response to lipoteichoic acid	2/114	14/18481	0.00327	Tirap/Cd14	2
BP	regulation of kinase activity	5/114	162/18481	0.003316	Mertk/Epha2/Cdk5rap1/Axl/Igf1r	5
BP	regulation of cell adhesion	3/114	50/18481	0.003629	Epha2/Serpine1/Tgfb2	3

	mediated by integrin					
BP	blood coagulation, fibrin clot formation	2/114	15/18481	0.003758	Apoh/F5	2
BP	myeloid leukocyte migration	6/114	243/18481	0.003979	Tirap/Serpine1/Jagn1/Tgfb2/Spp1/Il1r1	6
BP	substrate adhesion-dependent cell spreading	4/114	105/18481	0.004069	Fzd7/Mertk/Axl/Lamb3	4
BP	regulation of cell division	5/114	171/18481	0.004173	Cdc14a/Brca2/Sfn/Tgfb2/Igf1r	5
BP	regulation of transferase activity	5/114	171/18481	0.004173	Mertk/Epha2/Cdk5rap1/Axl/Igf1r	5
BP	nonribosomal peptide biosynthetic process	2/114	16/18481	0.004278	Ggt1/Aasdh	2
BP	response to cobalt ion	2/114	16/18481	0.004278	Alas1/Tigar	2
BP	somatic stem cell division	2/114	16/18481	0.004278	Fzd7/Tgfb2	2
BP	cellular response to ATP	2/114	16/18481	0.004278	Ptgs2/Serpine1	2
BP	tissue regeneration	4/114	107/18481	0.004352	Fzd7/Ifrd1/Serpine1/Axl	4
BP	cerebral cortex cell migration	3/114	55/18481	0.00475	Adgrg1/Lrp8/Mboat7	3
BP	response to laminar fluid shear stress	2/114	17/18481	0.004828	Serpine1/Tgfb2	2
BP	negative regulation of calcium ion transport	3/114	56/18481	0.004997	Ptgs2/Usp2/Tgfb2	3
BP	regulation of response to wounding	5/114	181/18481	0.0053	Apoh/Tnfrsf12a/Serpine1/Spp1/Igf1r	5
BP	regulation of amyloid-beta	2/114	18/18481	0.00541	Ttpa/Ifngr1	2

	clearance					
BP	biomineral tissue development	5/114	184/18481	0.005677	Ptgs2/Snx10/Gpnmb/Spp1/Bmp2k	5
BP	positive regulation of apoptotic signaling pathway	5/114	184/18481	0.005677	Daxx/Steap3/Tnfrsf12a/Tgfb2/Ripk3	5
BP	fibrinolysis	2/114	19/18481	0.006022	Apoh/Serpine1	2
BP	regulation of neuron migration	3/114	60/18481	0.006059	Adgrg1/Ntng2/Il1r1	3
BP	response to temperature stimulus	5/114	187/18481	0.006072	Daxx/Ptgs2/Il1r1/Tmem135/F3	5
BP	positive regulation of cell cycle	7/114	351/18481	0.006101	Cdc14a/Rad18/Brca2/Usp2/Tgfb2/Igf1r/Fam83d	7
BP	regulation of apoptotic signaling pathway	8/114	447/18481	0.006505	Daxx/Ptgs2/Steap3/Tnfrsf12a/Serpine1/Tgfb2/Cdkn2d/Ripk3	8
BP	myeloid leukocyte cytokine production	3/114	62/18481	0.006638	Tirap/Tgfb2/Axl	3
BP	female genitalia development	2/114	20/18481	0.006665	Mertk/Axl	2
BP	cellular response to mineralocorticoid stimulus	2/114	20/18481	0.006665	Serpine1/Igf1r	2
BP	protein activation cascade	2/114	20/18481	0.006665	Apoh/F5	2
BP	regulation of leukocyte apoptotic process	4/114	121/18481	0.006709	Mertk/Tgfb2/Ripk3/Axl	4
BP	response to angiotensin	3/114	64/18481	0.007247	Ptgs2/Serpine1/Igf1r	3
BP	positive regulation of endocytosis	5/114	198/18481	0.007686	Mfge8/Mertk/Serpine1/Axl/Cd14	5
BP	cellular response to testosterone	2/114	22/18481	0.008038	Spp1/Igf1r	2

	stimulus					
BP	cellular response to aldehyde	2/114	22/18481	0.008038	Serpine1/Igf1r	2
BP	type II interferon production	4/114	128/18481	0.008154	Ripk3/Axl/Il1r1/Cd14	4
BP	regulation of type II interferon production	4/114	128/18481	0.008154	Ripk3/Axl/Il1r1/Cd14	4
BP	regulation of cytokine production involved in immune response	4/114	130/18481	0.008601	Tirap/Tgfb2/Axl/Il1r1	4
BP	negative regulation of epithelial cell proliferation	5/114	205/18481	0.008856	Apoh/Brca2/Sfn/Tgfb2/Pex2	5
BP	cell-substrate adhesion	7/114	380/18481	0.009245	Fzd7/Mertk/Tnfrsf12a/Serpine1/Spp1/Axl/Lamb3	7
BP	telencephalon cell migration	3/114	70/18481	0.009272	Adgrg1/Lrp8/Mboat7	3
BP	cytokine production involved in immune response	4/114	133/18481	0.009302	Tirap/Tgfb2/Axl/Il1r1	4
BP	regulation of production of molecular mediator of immune response	5/114	210/18481	0.009764	Il13ra1/Tirap/Tgfb2/Axl/Il1r1	5
BP	tumor necrosis factor production	5/114	211/18481	0.009953	Tirap/Gpnmb/Ifngr1/Axl/Cd14	5
BP	regulation of tumor necrosis factor production	5/114	211/18481	0.009953	Tirap/Gpnmb/Ifngr1/Axl/Cd14	5
BP	negative regulation of axonogenesis	3/114	72/18481	0.010013	Ifrd1/Trak2/Spp1	3
BP	formation of primary germ layer	4/114	137/18481	0.010292	Fzd7/Epha2/Lamb3/Lama3	4
BP	positive regulation of	2/114	25/18481	0.010314	Apoh/Serpine1	2

	blood coagulation					
BP	regulation of collateral sprouting	2/114	25/18481	0.010314	Ifrd1/Spp1	2
BP	cellular response to arsenic-containing substance	2/114	25/18481	0.010314	Zfand2a/Daxx	2
BP	positive regulation of hemostasis	2/114	25/18481	0.010314	Apoh/Serpine1	2
BP	tumor necrosis factor superfamily cytokine production	5/114	214/18481	0.010535	Tirap/Gpnmb/Ifngr1/Axl/Cd14	5
BP	microtubule-based transport	5/114	214/18481	0.010535	Dync2i2/Trak2/Ssna1/Ap3m1/Dnaaf2	5
BP	regulation of tumor necrosis factor superfamily cytokine production	5/114	214/18481	0.010535	Tirap/Gpnmb/Ifngr1/Axl/Cd14	5
BP	leukocyte homeostasis	4/114	138/18481	0.01055	Mertk/Tgfb2/Ripk3/Axl	4
BP	negative regulation of muscle cell differentiation	3/114	74/18481	0.010787	Daxx/Fzd7/Rbpms2	3
BP	entrainment of circadian clock by photoperiod	2/114	26/18481	0.011129	Per1/Usp2	2
BP	cellular response to fluid shear stress	2/114	26/18481	0.011129	Ptgs2/Spp1	2
BP	cellular response to interleukin-1	4/114	141/18481	0.011349	Rbmx/Serpine1/Tank/Il1r1	4
BP	female pregnancy	6/114	304/18481	0.011439	Slc19a1/Ptgs2/Serpine1/Tgfb2/Timp1/Hsd17b7	6
BP	positive regulation of cell	3/114	76/18481	0.011595	Cdc14a/Tgfb2/Igf1r	3

	division					
BP	leukocyte migration	7/114	399/18481	0.011866	Tirap/Serpine1/Jagn1/Tgfb2/Spp1/Ripk3/Il1r1	7
BP	plasminogen activation	2/114	27/18481	0.011972	Apoh/Serpine1	2
BP	iron ion transmembrane transport	2/114	27/18481	0.011972	Steap3/Slc39a14	2
BP	response to hypoxia	8/114	499/18481	0.012156	Daxx/Ptgs2/Alas1/Serpine1/Tgfb2/Sdc2/Tigar/Arnt2	8
BP	phosphatidylinositol metabolic process	4/114	144/18481	0.012186	Ttc7b/Mboat7/Lpgat1/Pigb	4
BP	regulation of toll-like receptor 4 signaling pathway	2/114	28/18481	0.012841	Tirap/Cd14	2
BP	positive regulation of coagulation	2/114	28/18481	0.012841	Apoh/Serpine1	2
BP	cellular response to glucose stimulus	5/114	226/18481	0.013096	Serpine1/Jagn1/Il1r1/Igf1r/Slc39a14	5
BP	positive regulation of lipid metabolic process	4/114	148/18481	0.013361	Apoh/Ptgs2/Lpgat1/Igf1r	4
BP	cellular response to hexose stimulus	5/114	228/18481	0.01356	Serpine1/Jagn1/Il1r1/Igf1r/Slc39a14	5
BP	anatomical structure maturation	6/114	316/18481	0.013632	Nfasc/Anks1a/Brca2/Snx10/Tgfb2/Axl	6
BP	positive regulation of fatty acid biosynthetic process	2/114	29/18481	0.013737	Ptgs2/Lpgat1	2
BP	negative regulation of epithelial cell apoptotic process	3/114	82/18481	0.014224	Ttpa/Serpine1/Igf1r	3

BP	cellular response to monosaccharide stimulus	5/114	231/18481	0.014278	Serpine1/Jagn1/Il1r1/Igf1r/Slc39a14	5
BP	negative regulation of angiogenesis	4/114	151/18481	0.014289	Apoh/Epha2/Serpine1/Tgfb2	4
BP	entrainment of circadian clock	2/114	30/18481	0.01466	Per1/Usp2	2
BP	regulation of hair cycle	2/114	30/18481	0.01466	Per1/Tgfb2	2
BP	leukocyte apoptotic process	4/114	153/18481	0.01493	Mertk/Tgfb2/Ripk3/Axl	4
BP	negative regulation of blood vessel morphogenesis	4/114	153/18481	0.01493	Apoh/Epha2/Serpine1/Tgfb2	4
BP	regulation of cytokinesis	3/114	84/18481	0.01517	Cdc14a/Brca2/Igf1r	3
BP	negative regulation of vasculature development	4/114	154/18481	0.015257	Apoh/Epha2/Serpine1/Tgfb2	4
BP	substrate-dependent cell migration	2/114	31/18481	0.015608	Anks1a/Tnfrsf12a	2
BP	positive regulation of extracellular matrix organization	2/114	31/18481	0.015608	Ier3ip1/Tgfb2	2
BP	positive regulation of response to wounding	3/114	85/18481	0.015655	Apoh/Serpine1/Igf1r	3
BP	multi-organism reproductive process	6/114	327/18481	0.015886	Slc19a1/Ptgs2/Serpine1/Tgfb2/Timp1/Hsd17b7	6
BP	reproductive structure development	7/114	424/18481	0.016086	Adgrg1/Brca2/Mertk/Serpine1/Tgfb2/Axl/Igf1r	7
BP	response to purine-containing	5/114	239/18481	0.016314	Per1/Ptgs2/Alas1/Serpine1/Sdc2	5

	compound					
BP	photoperiodism	2/114	32/18481	0.016582	Per1/Usp2	2
BP	mammary gland epithelial cell proliferation	2/114	32/18481	0.016582	Brca2/Epha2	2
BP	cell adhesion mediated by integrin	3/114	87/18481	0.016653	Epha2/Serpine1/Tgfb2	3
BP	response to hexose	6/114	331/18481	0.016766	Ptgs2/Serpine1/Jagn1/Il1r1/Igf1r/Slc39a14	6
BP	phosphatidylinositol 3-kinase/protein kinase B signal transduction	6/114	332/18481	0.016992	Mertk/Epha2/Tgfb2/Axl/F3/Igf1r	6
BP	reproductive system development	7/114	429/18481	0.017044	Adgrg1/Brca2/Mertk/Serpine1/Tgfb2/Axl/Igf1r	7
BP	gliogenesis	7/114	430/18481	0.01724	Adgrg1/Lrp8/Ifrd1/Tgfb2/Ifngr1/Mboat7/Nap1l1	7
BP	neutrophil homeostasis	2/114	33/18481	0.017582	Mertk/Axl	2
BP	neuron projection arborization	2/114	33/18481	0.017582	Ntng2/Ssna1	2
BP	response to aldosterone	2/114	33/18481	0.017582	Serpine1/Igf1r	2
BP	intracellular glucose homeostasis	5/114	244/18481	0.01768	Serpine1/Jagn1/Il1r1/Igf1r/Slc39a14	5
BP	stress-activated MAPK cascade	5/114	244/18481	0.01768	Per1/Daxx/Tirap/Tgfb2/Igf1r	5
BP	cellular response to abiotic stimulus	7/114	435/18481	0.018245	Ptgs2/Brca2/Serpine1/Timp1/Tank/Mcoln1/Igf1r	7
BP	cellular response to environmental stimulus	7/114	435/18481	0.018245	Ptgs2/Brca2/Serpine1/Timp1/Tank/Mcoln1/Igf1r	7
BP	cellular response to carbohydrate stimulus	5/114	246/18481	0.018247	Serpine1/Jagn1/Il1r1/Igf1r/Slc39a14	5
BP	natural killer cell differentiation	2/114	34/18481	0.018606	Mertk/Axl	2

BP	regulation of cardiocyte differentiation	2/114	34/18481	0.018606	Fzd7/Tgfb2	2
BP	JNK cascade	4/114	164/18481	0.018777	Per1/Daxx/Tirap/Igf1r	4
BP	response to UV	4/114	164/18481	0.018777	Rad18/Brca2/Timp1/Cdkn2d	4
BP	protein processing	5/114	248/18481	0.018826	F9/Apoh/Ggt1/Serpine1/F3	5
BP	hormone metabolic process	5/114	248/18481	0.018826	Spp1/Hsd17b7/Retsat/Dhrs9/Igf1r	5
BP	multi-multicellular organism process	6/114	341/18481	0.019114	Slc19a1/Ptgs2/Serpine1/Tgfb2/Timp1/Hsd17b7	6
BP	regulation of immune effector process	7/114	440/18481	0.019291	RT1-DMb/Il13ra1/Tirap/Tgfb2/Ripk3/Axl/Il1r1	7
BP	negative regulation of response to wounding	3/114	92/18481	0.0193	Apoh/Serpine1/Spp1	3
BP	steroid metabolic process	6/114	342/18481	0.01936	Timp1/Spp1/Hsd17b7/Dhrs9/Igf1r/Pex2	6
BP	collateral sprouting	2/114	35/18481	0.019656	Ifrd1/Spp1	2
BP	production of molecular mediator of immune response	5/114	252/18481	0.020021	Il13ra1/Tirap/Tgfb2/Axl/Il1r1	5
BP	maternal process involved in female pregnancy	3/114	94/18481	0.02042	Ptgs2/Serpine1/Hsd17b7	3
BP	cytokinesis	4/114	169/18481	0.020711	Cdc14a/Daxx/Brca2/Igf1r	4
BP	negative regulation of neuron projection development	4/114	169/18481	0.020711	Ifrd1/Trak2/Spp1/Crmp1	4
BP	negative regulation of cytokine production	2/114	36/18481	0.020729	Tgfb2/Axl	2

	involved in immune response					
BP	response to monosaccharide	6/114	348/18481	0.020885	Ptgs2/Serpine1/Jagn1/Il1r1/Igf1r/Slc39a14	6
BP	stress-activated protein kinase signaling cascade	5/114	255/18481	0.020948	Per1/Daxx/Tirap/Tgfb2/Igf1r	5
BP	calcium ion transport	7/114	451/18481	0.02174	Tpcn2/Ptgs2/Atp2c1/Usp2/Serpine1/Tgfb2/Mcoln1	7
BP	positive regulation of cytokinesis	2/114	37/18481	0.021826	Cdc14a/Igf1r	2
BP	transport along microtubule	4/114	172/18481	0.021928	Dync2i2/Trak2/Ssna1/Ap3m1	4
BP	selective autophagy	3/114	97/18481	0.022166	Map1lc3b/Tigar/Pex2	3
BP	regulation of axon regeneration	2/114	38/18481	0.022947	Spp1/Igf1r	2
BP	steroid biosynthetic process	4/114	175/18481	0.023188	Timp1/Hsd17b7/Igf1r/Pex2	4
BP	calcium ion homeostasis	6/114	357/18481	0.023321	Tpcn2/Atp2c1/Snx10/Tgfb2/Spp1/Mcoln1	6
BP	phosphatidylinositol biosynthetic process	3/114	99/18481	0.023374	Ttc7b/Mboat7/Pigb	3
BP	regulation of lymphocyte proliferation	5/114	263/18481	0.023558	RT1-DMb/Il13ra1/Tirap/Gpnmb/Ripk3	5
BP	response to interleukin-1	4/114	176/18481	0.023618	Rbmx/Serpine1/Tank/Il1r1	4
BP	peroxisome organization	2/114	39/18481	0.024092	Tmem135/Pex2	2
BP	protein targeting to ER	2/114	39/18481	0.024092	Tram1/Zfand2a	2
BP	decidualization	2/114	39/18481	0.024092	Ptgs2/Serpine1	2
BP	ephrin receptor signaling pathway	2/114	39/18481	0.024092	Anks1a/Epha2	2
BP	regulation of intrinsic	2/114	39/18481	0.024092	Steap3/Cdkn2d	2

	apoptotic signaling pathway in response to DNA damage					
BP	macroautophagy	5/114	265/18481	0.024242	Supt5h/Map1lc3b/Mcoln1/Tigar/Pex2	5
BP	neutrophil chemotaxis	3/114	101/18481	0.024617	Tirap/Tgfb2/Spp1	3
BP	pallium development	5/114	267/18481	0.024938	Adgrg1/Lrp8/Hsd17b7/Mboat7/Igf1r	5
BP	regulation of mononuclear cell proliferation	5/114	267/18481	0.024938	RT1-DMb/Il13ra1/Tirap/Gpnmb/Ripk3	5
BP	response to light stimulus	6/114	363/18481	0.025048	Per1/Rad18/Brca2/Usp2/Timp1/Cdkn2d	6
BP	negative regulation of blood coagulation	2/114	40/18481	0.025259	Apoh/Serpine1	2
BP	cellular response to metal ion	5/114	268/18481	0.025292	Daxx/Ptgs2/Serpine1/Mcoln1/Tigar	5
BP	regulation of lipid metabolic process	6/114	364/18481	0.025344	Apoh/Ptgs2/Mboat7/Lpgat1/Igf1r/Pex2	6
BP	regulation of angiogenesis	6/114	364/18481	0.025344	Apoh/Epha2/Tnfrsf12a/Serpine1/Tgfb2/F3	6
BP	negative regulation of epithelial to mesenchymal transition	2/114	41/18481	0.026449	Sdhaf2/Tgfb2	2
BP	response to ATP	2/114	41/18481	0.026449	Ptgs2/Serpine1	2
BP	negative regulation of hemostasis	2/114	41/18481	0.026449	Apoh/Serpine1	2
BP	regulation of epithelial cell proliferation	7/114	471/18481	0.026732	Apoh/Brca2/Fzd7/Sfn/Tgfb2/F3/Pex2	7
BP	regulation of vasculature development	6/114	369/18481	0.026859	Apoh/Epha2/Tnfrsf12a/Serpine1/Tgfb2/F3	6
BP	mitophagy	2/114	42/184	0.02766	Map1lc3b/Tigar	2

			81	1		
BP	regulation of neuron projection regeneration	2/114	42/18481	0.027661	Spp1/Igf1r	2
BP	cellular response to low-density lipoprotein particle stimulus	2/114	42/18481	0.027661	Serpine1/F3	2
BP	positive regulation of neutrophil migration	2/114	42/18481	0.027661	Tirap/Il1r1	2
BP	response to starvation	5/114	275/18481	0.027853	Map1lc3b/Pdp2/Serpine1/Igf1r/Pex2	5
BP	response to heat	3/114	106/18481	0.027879	Daxx/Ptgs2/Il1r1	3
BP	cardiac atrium development	2/114	43/18481	0.028896	Tgfb2/Igf1r	2
BP	response to lipoprotein particle	2/114	43/18481	0.028896	Serpine1/F3	2
BP	establishment of protein localization to endoplasmic reticulum	2/114	43/18481	0.028896	Tram1/Zfand2a	2
BP	female sex differentiation	4/114	189/18481	0.029646	Brca2/Mertk/Serpine1/Axl	4
BP	regulation of axonogenesis	4/114	189/18481	0.029646	Ifrd1/Tnfrsf12a/Trak2/Spp1	4
BP	stem cell division	2/114	44/18481	0.030152	Fzd7/Tgfb2	2
BP	intrinsic apoptotic signaling pathway in response to DNA damage by p53 class mediator	2/114	44/18481	0.030152	Steap3/Brca2	2
BP	negative regulation of coagulation	2/114	44/18481	0.030152	Apoh/Serpine1	2
BP	negative regulation of	2/114	44/18481	0.030152	Daxx/Fzd7	2

	striated muscle cell differentiation					
BP	regulation of transforming growth factor beta production	2/114	44/18481	0.030152	Ptgs2/Tgfb2	2
BP	regulation of phosphatidylinositol 3-kinase/protein kinase B signal transduction	5/114	281/18481	0.030176	Epha2/Tgfb2/Axl/F3/Igf1r	5
BP	positive regulation of lipid biosynthetic process	3/114	110/18481	0.030646	Ptgs2/Lpgat1/Igf1r	3
BP	membrane protein ectodomain proteolysis	2/114	45/18481	0.031429	Rbmx/Timp1	2
BP	intracellular pH reduction	2/114	45/18481	0.031429	Tpcn2/Ttpa	2
BP	bone remodeling	3/114	112/18481	0.032082	Epha2/Snx10/Spp1	3
BP	regulation of mitotic cell cycle	7/114	490/18481	0.032154	Anapc15/Cdc14a/Brca2/Usp2/Cdkn2d/Gpnmb/Igf1r	7
BP	positive regulation of phosphatidylinositol 3-kinase/protein kinase B signal transduction	4/114	194/18481	0.032186	Tgfb2/Axl/F3/Igf1r	4
BP	developmental maturation	6/114	386/18481	0.032456	Nfasc/Anks1a/Brca2/Snx10/Tgfb2/Axl	6
BP	intraciliary transport	2/114	46/18481	0.032728	Dync2i2/Ssna1	2
BP	transforming growth factor beta production	2/114	46/18481	0.032728	Ptgs2/Tgfb2	2
BP	regulation of catalytic activity	7/114	492/18481	0.032765	Mertk/Epha2/Serpine1/Timp1/Cdk5rap1/A	7

					xl/Igf1r	
BP	response to carbohydrate	6/114	388/18481	0.033161	Ptgs2/Serpine1/Jagn1/Il1r1/Igf1r/Slc39a14	6
BP	regulation of leukocyte proliferation	5/114	289/18481	0.033459	RT1-DMb/Il13ra1/Tirap/Gpnmb/Ripk3	5
BP	regulation of stress-activated MAPK cascade	4/114	197/18481	0.03377	Per1/Tirap/Tgfb2/Igf1r	4
BP	regulation of cellular extravasation	2/114	47/18481	0.034047	Ripk3/Il1r1	2
BP	vesicle transport along microtubule	2/114	47/18481	0.034047	Trak2/Ap3m1	2
BP	canonical NF-kappaB signal transduction	5/114	292/18481	0.034745	Per1/Tirap/Atp2c1/Tank/Ripk3	5
BP	positive regulation of B cell proliferation	2/114	48/18481	0.035387	Il13ra1/Tirap	2
BP	cellular response to lipoprotein particle stimulus	2/114	48/18481	0.035387	Serpine1/F3	2
BP	homeostasis of number of cells	6/114	395/18481	0.035707	Adgrg1/Mertk/Alas1/Tgfb2/Ripk3/Axl	6
BP	negative regulation of cellular catabolic process	4/114	201/18481	0.035951	Tirap/Tent5d/Timp1/Tigar	4
BP	regulation of stress-activated protein kinase signaling cascade	4/114	201/18481	0.035951	Per1/Tirap/Tgfb2/Igf1r	4
BP	cytoskeleton-dependent intracellular transport	4/114	202/18481	0.036509	Dync2i2/Trak2/Ssna1/Ap3m1	4
BP	response to organophosphorus	4/114	202/18481	0.036509	Per1/Ptgs2/Alas1/Serpine1	4
BP	negative regulation of production of	2/114	49/18481	0.036747	Tgfb2/Axl	2

	molecular mediator of immune response					
BP	endodermal cell differentiation	2/114	49/18481	0.036747	Lamb3/Lama3	2
BP	response to arsenic-containing substance	2/114	49/18481	0.036747	Zfand2a/Daxx	2
BP	sex differentiation	6/114	398/18481	0.036835	Adgrg1/Brca2/Mertk/Serpine1/Tgfb2/Axl	6
BP	triglyceride metabolic process	3/114	119/18481	0.037381	Apoh/Mboat7/Lpgat1	3
BP	negative regulation of cell growth	4/114	204/18481	0.037639	Ifrd1/Tgfb2/Cdkn2d/Spp1	4
BP	regulation of type I interferon production	3/114	120/18481	0.038172	Tirap/Tank/Cd14	3
BP	type I interferon production	3/114	120/18481	0.038172	Tirap/Tank/Cd14	3
BP	negative regulation of monoatomic ion transport	3/114	120/18481	0.038172	Ptgs2/Usp2/Tgfb2	3
BP	regulation of intrinsic apoptotic signaling pathway	4/114	205/18481	0.038212	Ptgs2/Steap3/Cdkn2d/Ripk3	4
BP	positive regulation of tumor necrosis factor production	3/114	121/18481	0.038972	Tirap/Ifngr1/Cd14	3
BP	response to tumor necrosis factor	5/114	302/18481	0.039253	Daxx/Ptgs2/Ggt1/Tank/Cd14	5
BP	positive regulation of fatty acid metabolic process	2/114	51/18481	0.039527	Ptgs2/Lpgat1	2
BP	vitamin transport	2/114	51/18481	0.039527	Slc19a1/Ttpa	2

BP	transition metal ion transport	3/114	122/18481	0.03978	Steap3/Atp2c1/Slc39a14	3
BP	positive regulation of tumor necrosis factor superfamily cytokine production	3/114	123/18481	0.040597	Tirap/Ifngr1/Cd14	3
BP	maternal placenta development	2/114	52/18481	0.040945	Ptgs2/Serpine1	2
BP	toll-like receptor 4 signaling pathway	2/114	52/18481	0.040945	Tirap/Cd14	2
BP	retinol metabolic process	2/114	52/18481	0.040945	Retsat/Dhrs9	2
BP	genitalia development	2/114	52/18481	0.040945	Mertk/Axl	2
BP	regulation of T cell apoptotic process	2/114	52/18481	0.040945	Tgfb2/Ripk3	2
BP	regulation of neutrophil migration	2/114	52/18481	0.040945	Tirap/Il1r1	2
BP	regulation of muscle cell apoptotic process	3/114	125/18481	0.042256	Apoh/Tigar/Igf1r	3
BP	anterograde axonal transport	2/114	53/18481	0.042383	Trak2/Ap3m1	2
BP	regulation of developmental growth	6/114	413/18481	0.042821	Daxx/Ifrd1/Tnfrsf12a/Serpine1/Spp1/Igf1r	6
BP	glycerolipid biosynthetic process	4/114	213/18481	0.042975	Ttc7b/Mboat7/Lpgat1/Pigb	4
BP	granulocyte chemotaxis	3/114	126/18481	0.043099	Tirap/Tgfb2/Spp1	3
BP	regulation of epithelial cell migration	5/114	310/18481	0.043105	Apoh/Ptgs2/Epha2/Serpine1/Tgfb2	5
BP	cilium organization	6/114	415/18481	0.043663	Cdc14a/Dync2i2/Snx10/Ssna1/Bbs9/Dnaaf2	6
BP	T cell	2/114	54/184	0.04384	Tgfb2/Ripk3	2

	homeostasis		81			
BP	autophagosome maturation	2/114	54/18481	0.04384	Map1lc3b/Mcoln1	2
BP	gastrulation	4/114	215/18481	0.044216	Fzd7/Epha2/Lamb3/Lama3	4
BP	regulation of lipid biosynthetic process	4/114	216/18481	0.044843	Ptgs2/Lpgat1/Igf1r/Pex2	4
BP	regulation of triglyceride metabolic process	2/114	55/18481	0.045315	Apoh/Mboat7	2
BP	cellular response to angiotensin	2/114	55/18481	0.045315	Serpine1/Igf1r	2
BP	monocarboxylic acid biosynthetic process	4/114	217/18481	0.045476	Ptgs2/Lpgat1/Dhrs9/Pex2	4
BP	negative regulation of canonical NF-kappaB signal transduction	2/114	56/18481	0.046808	Per1/Tank	2
BP	response to glucose	5/114	318/18481	0.047178	Serpine1/Jagn1/Il1r1/Igf1r/Slc39a14	5
BP	carboxylic acid biosynthetic process	5/114	318/18481	0.047178	Ptgs2/Ggt1/Lpgat1/Dhrs9/Pex2	5
BP	molting cycle	3/114	131/18481	0.047436	Per1/Ptgs2/Tgfb2	3
BP	hair cycle	3/114	131/18481	0.047436	Per1/Ptgs2/Tgfb2	3
BP	positive regulation of growth	5/114	319/18481	0.047703	Sfn/Tnfrsf12a/Serpine1/Tgfb2/Igf1r	5
BP	cell chemotaxis	5/114	320/18481	0.048231	Tirap/Epha2/Serpine1/Tgfb2/Spp1	5
BP	regulation of fatty acid biosynthetic process	2/114	57/18481	0.048319	Ptgs2/Lpgat1	2
BP	skeletal muscle tissue regeneration	2/114	57/18481	0.048319	Fzd7/Ifrd1	2
BP	icosanoid	2/114	57/184	0.04831	Ptgs2/Ggt1	2

	biosynthetic process		81	9		
BP	intracellular protein transmembrane transport	2/114	57/18481	0.048319	Tram1/Pex2	2
BP	regulation of JNK cascade	3/114	132/18481	0.048328	Per1/Tirap/Igf1r	3
BP	regulation of wound healing	3/114	132/18481	0.048328	Apoh/Tnfrsf12a/Serpine1	3
BP	regulation of extent of cell growth	3/114	132/18481	0.048328	Ifrd1/Tnfrsf12a/Spp1	3
BP	negative regulation of catalytic activity	3/114	133/18481	0.049229	Serpine1/Timp1/Cdk5rap1	3
BP	response to axon injury	3/114	133/18481	0.049229	Spp1/Axl/Igf1r	3
BP	release of sequestered calcium ion into cytosol	3/114	133/18481	0.049229	Tpcn2/Tgfb2/Mcoln1	3
BP	organic acid biosynthetic process	5/114	322/18481	0.049298	Ptgs2/Ggt1/Lpgat1/Dhrs9/Pex2	5
BP	protein localization to microtubule cytoskeleton	2/114	58/18481	0.049848	Snx10/Fam83d	2
CC	endosome membrane	9/121	400/18710	0.001172	RT1-DMb/Tpcn2/Steap3/Fzd7/Micall1/Snx10/Gpnmb/Mcoln1/Slc39a14	9
CC	laminin complex	2/121	11/18710	0.002196	Lamb3/Lama3	2
CC	receptor complex	8/121	402/18710	0.004629	Lrp8/Mertk/Epha2/Mcoln1/Axl/Igf1r/Arnt2/Cd14	8
CC	basement membrane	4/121	109/18710	0.005494	Tgfb2/Timp1/Lamb3/Lama3	4
CC	melanosome membrane	2/121	18/18710	0.005931	Tpcn2/Gpnmb	2
CC	chitosome	2/121	18/187	0.00593	Tpcn2/Gpnmb	2

			10	1		
CC	pigment granule membrane	2/121	18/18710	0.005931	Tpcn2/Gpnmb	2
CC	collagen-containing extracellular matrix	6/121	256/18710	0.006429	Serpine1/Tgfb2/Timp1/Lamb3/F3/Lama3	6
CC	XY body	2/121	23/18710	0.009603	Daxx/Rad18	2
CC	late endosome	6/121	281/18710	0.009933	RT1-DMb/Steap3/Ttpa/Micall1/Mcoln1/Slc39a14	6
CC	nuclear outer membrane	2/121	28/18710	0.014049	Ptgs2/Retsat	2
CC	secretory granule	7/121	445/18710	0.025638	F5/Brca2/Atp2c1/Mfge8/Snx10/Serpine1/Tgfb2	7
CC	melanosome	3/121	101/18710	0.027807	Tpcn2/Gpnmb/Nap1l1	3
CC	pigment granule	3/121	101/18710	0.027807	Tpcn2/Gpnmb/Nap1l1	3
CC	sex chromosome	2/121	41/18710	0.028864	Daxx/Rad18	2
CC	caveola	3/121	104/18710	0.029969	Lrp8/Ptgs2/Igf1r	3
CC	ciliary basal body	4/121	181/18710	0.030048	Cdc14a/Dync2i2/Ttll4/Ssna1	4
CC	nuclear ubiquitin ligase complex	2/121	42/18710	0.030182	Anapc15/Brca2	2
CC	glial cell projection	2/121	46/18710	0.035682	Nfasc/Adgrg1	2
CC	plasma membrane bounded cell projection cytoplasm	5/121	282/18710	0.036403	Dync2i2/Map1lc3b/Trak2/Ssna1/Ap3m1	5
CC	transcription elongation factor complex	2/121	49/18710	0.040041	Supt5h/Pex2	2
CC	peptidase complex	3/121	117/18710	0.040329	Zfand2a/Psmc3/F3	3
CC	actin cytoskeleton	7/121	500/18710	0.043806	Gas2l3/Ocm/Nexn/Hnrnpk-	7

					ps1/Tagln3/Crmp1/Axl	
CC	extracellular matrix	6/121	398/18710	0.044719	Serpine1/Tgfb2/Timp1/Lamb3/F3/Lama3	6
CC	external encapsulating structure	6/121	400/18710	0.045627	Serpine1/Tgfb2/Timp1/Lamb3/F3/Lama3	6
CC	outer membrane	4/121	209/18710	0.046868	Ptgs2/Retsat/Tigar/Cyb5b	4
CC	organelle outer membrane	4/121	209/18710	0.046868	Ptgs2/Retsat/Tigar/Cyb5b	4
MF	phospholipid binding	12/107	486/17065	5.30E-05	Apoh/Tpcn2/Anxa10/Tirap/Map1lc3b/Fzd7/Ttpa/Mfge8/Micall1/Snx10/Axl/F3	12
MF	kinesin binding	4/107	54/17065	0.000363	Lrp8/Daxx/Trak2/Fam83d	4
MF	transmembrane receptor protein tyrosine kinase activity	4/107	58/17065	0.000478	Mertk/Epha2/Axl/Igf1r	4
MF	transmembrane receptor protein kinase activity	4/107	75/17065	0.001262	Mertk/Epha2/Axl/Igf1r	4
MF	sugar-phosphatase activity	2/107	12/17065	0.002467	Tigar/Pfkfb4	2
MF	cytokine receptor activity	4/107	94/17065	0.002891	Il13ra1/Ifngr1/Il1r1/F3	4
MF	phosphatidylethanolamine binding	2/107	13/17065	0.002904	Map1lc3b/Mfge8	2
MF	carbohydrate phosphatase activity	2/107	13/17065	0.002904	Tigar/Pfkfb4	2
MF	protease binding	5/107	156/17065	0.003011	Brca2/Serpine1/Timp1/Il1r1/F3	5
MF	calcium-release channel activity	2/107	17/17065	0.004981	Tpcn2/Mcoln1	2
MF	phosphatidylinositol bisphosphate binding	4/107	114/17065	0.005751	Tpcn2/Tirap/Fzd7/Ttpa	4
MF	phosphatidylinositol phosphate binding	5/107	189/17065	0.006762	Tpcn2/Tirap/Fzd7/Ttpa/Snx10	5

MF	lysophospholipid acyltransferase activity	2/107	20/17065	0.006875	Mboat7/Lpgat1	2
MF	protein tyrosine kinase activity	4/107	128/17065	0.008603	Mertk/Epha2/Axl/Igf1r	4
MF	immune receptor activity	4/107	132/17065	0.009561	Il13ra1/Ifngr1/Il1r1/F3	4
MF	ligand-gated calcium channel activity	2/107	26/17065	0.011476	Tpcn2/Mcoln1	2
MF	hydrolase activity, acting on carbon-nitrogen (but not peptide) bonds, in cyclic amidines	2/107	27/17065	0.012343	Adat1/Mthfd2	2
MF	growth factor binding	4/107	144/17065	0.012843	Epha2/Axl/Il1r1/Igf1r	4
MF	intracellular ligand-gated monoatomic ion channel activity	2/107	29/17065	0.014162	Tpcn2/Mcoln1	2
MF	ubiquitin protein ligase binding	6/107	318/17065	0.015023	Per1/Daxx/Rad18/Map1lc3b/Usp2/Tank	6
MF	cell-cell adhesion mediator activity	2/107	31/17065	0.016089	Nfasc/Nexn	2
MF	phosphatidylinositol-4,5-bisphosphate binding	3/107	88/17065	0.017896	Tirap/Fzd7/Ttpa	3
MF	ubiquitin-like protein ligase binding	6/107	336/17065	0.019167	Per1/Daxx/Rad18/Map1lc3b/Usp2/Tank	6
MF	modified amino acid binding	3/107	91/17065	0.019548	Slc19a1/Mfge8/Axl	3
MF	steroid dehydrogenase activity, acting on the CH-OH group of donors, NAD or NADP as acceptor	2/107	35/17065	0.020256	Hsd17b7/Dhrs9	2
MF	phosphoric ester hydrolase	6/107	355/17065	0.024327	Hddc3/Cdc14a/Pdp2/Ier3ip1/Tigar/Pfkfb4	6

	activity					
MF	phosphatidylinositol 3-kinase binding	2/107	39/17065	0.024822	Axl/Igf1r	2
MF	heme binding	4/107	178/17065	0.025755	Ptgs2/Steap3/Cyp4v3/Cyb5b	4
MF	transition metal ion transmembrane transporter activity	2/107	40/17065	0.026023	Atp2c1/Slc39a14	2
MF	phosphatase activity	5/107	270/17065	0.027568	Cdc14a/Pdp2/Ier3ip1/Tigar/Pfkfb4	5
MF	steroid dehydrogenase activity	2/107	43/17065	0.029764	Hsd17b7/Dhrs9	2
MF	cell adhesion mediator activity	2/107	43/17065	0.029764	Nfasc/Nexn	2
MF	tetrapyrrole binding	4/107	187/17065	0.030126	Ptgs2/Steap3/Cyp4v3/Cyb5b	4
MF	phosphatidylinositol binding	5/107	283/17065	0.032808	Tpcn2/Tirap/Fzd7/Ttpa/Snx10	5
MF	tubulin binding	6/107	388/17065	0.035361	Gas2l3/Brca2/Map1lc3b/Ttll4/Ssna1/Fam83d	6
MF	cell adhesion molecule binding	5/107	290/17065	0.035871	Nfasc/Nexn/Mfge8/Gpnmb/Spp1	5
MF	amide binding	6/107	397/17065	0.038852	Lrp8/Slc19a1/Map1lc3b/Tgfb2/Tpp2/Igf1r	6
MF	O-acyltransferase activity	2/107	54/17065	0.045128	Mboat7/Lpgat1	2
MF	hydrolase activity, acting on carbon-nitrogen (but not peptide) bonds	3/107	128/17065	0.046599	Adat1/Crmp1/Mthfd2	3
