# Supplementary material for: The Altered Proteomic Landscape in Renal Tubular Epithelial Cells under High Oxalate Stimulation
Source: Biology (Basel). 2024 Oct 11;13(10):814. doi: 10.3390/biology13100814 (PMC11505525; doi:10.3390/biology13100814)
Supplement: Supplementary file 1 [file biology-13-00814-s001.zip › Table S3.pdf]

Table S3. The detailed results of GO enrichment analysis of the down-regulated DEPs.

Category	Description	Protein Ratio	Bg Ratio	P-value	Protein ID	Count
BP	positive regulation of monocyte differentiation	3/115	11/18481	3.73E-05	Cd74/Jun/Zfp36l1	3
BP	regulation of DNA-templated transcription initiation	5/115	68/18481	6.52E-05	Taf4/Sub1/Taf3/Thra/Jun	5
BP	regulation of monocyte differentiation	3/115	21/18481	0.000288	Cd74/Jun/Zfp36l1	3
BP	regulation of myeloid leukocyte differentiation	6/115	147/18481	0.000316	Cd74/Jun/Zfpm1/Zfp36l1/Pias3/Fos	6
BP	positive regulation of DNA-templated transcription initiation	4/115	57/18481	0.000436	Taf4/Sub1/Taf3/Jun	4
BP	regulation of transcription initiation by RNA polymerase II	4/115	59/18481	0.000497	Taf4/Sub1/Taf3/Thra	4
BP	cellular response to nutrient levels	8/115	307/18481	0.000673	Rnf167/Foxo1/Otud3/Rragd/Jun/Gabarap/Eln/Fos	8
BP	RNA polymerase II preinitiation complex assembly	3/115	32/18481	0.001021	Taf4/Taf3/Thra	3
BP	DNA-templated transcription initiation	5/115	124/18481	0.001069	Taf4/Sub1/Taf3/Thra/Jun	5
BP	cellular response to extracellular stimulus	8/115	344/18481	0.001398	Rnf167/Foxo1/Otud3/Rragd/Jun/Gabarap/Eln/Fos	8
BP	positive regulation of	4/115	79/18481	0.001493	Cd74/Jun/Zfp36l1/Fos	4

	myeloid leukocyte differentiation					
BP	monocyte differentiation	3/115	37/18481	0.001563	Cd74/Jun/Zfp36l1	3
BP	myeloid leukocyte differentiation	7/115	273/18481	0.001605	Tlr4/Cd74/Jun/Zfpm1/Zfp36l1/Pias3/Fos	7
BP	transcription initiation at RNA polymerase II promoter	4/115	83/18481	0.001791	Taf4/Sub1/Taf3/Thra	4
BP	neuron migration	6/115	209/18481	0.001971	Col3a1/Dclk1/Adgrl3/Arhgap32/Fgf13/Phactr1	6
BP	cellular response to starvation	6/115	211/18481	0.002068	Rnf167/Foxo1/Rragd/Jun/Gabarap/Fos	6
BP	regulation of vitamin metabolic process	2/115	12/18481	0.002432	Rdh10/Cd320	2
BP	elastic fiber assembly	2/115	12/18481	0.002432	Col3a1/Eln	2
BP	response to prolactin	2/115	12/18481	0.002432	Jun/Fos	2
BP	transcription preinitiation complex assembly	3/115	44/18481	0.002582	Taf4/Taf3/Thra	3
BP	keratinocyte differentiation	5/115	152/18481	0.002618	Tgm1/Zfp36l1/Krt5/Krt10/Krt2	5
BP	cellular response to external stimulus	9/115	470/18481	0.002685	Tlr4/Rnf167/Foxo1/Otud3/Rragd/Jun/Gabarap/Eln/Fos	9
BP	MyD88-independent toll-like receptor signaling pathway	2/115	13/18481	0.002863	Tlr4/Rab11fip2	2
BP	cellular response to leucine starvation	2/115	13/18481	0.002863	Rnf167/Rragd	2
BP	regulation of	4/115	95/184	0.002931	Ctcf/Jun/Dedd/Fos	4

	ncRNA transcription		81			
BP	mRNA transcription by RNA polymerase II	3/115	46/18481	0.002932	Taf4/Taf3/Thra	3
BP	positive regulation of lymphocyte proliferation	5/115	157/18481	0.00301	Tlr4/Cd74/Vtcn1/Efnb1/Cd320	5
BP	positive regulation of mononuclear cell proliferation	5/115	160/18481	0.003264	Tlr4/Cd74/Vtcn1/Efnb1/Cd320	5
BP	protein-DNA complex assembly	5/115	160/18481	0.003264	Taf4/Pias1/Taf3/H3f3b/Thra	5
BP	positive regulation of B cell proliferation	3/115	48/18481	0.003311	Tlr4/Cd74/Cd320	3
BP	positive regulation of transcription initiation by RNA polymerase II	3/115	50/18481	0.003719	Taf4/Sub1/Taf3	3
BP	positive regulation of chemokine (C-X-C motif) ligand 2 production	2/115	15/18481	0.003823	Tlr4/Cd74	2
BP	regulation of myeloid cell differentiation	6/115	240/18481	0.003909	Cd74/Jun/Zfpm1/Zfp36l1/Pias3/Fos	6
BP	mRNA transcription	3/115	51/18481	0.003933	Taf4/Taf3/Thra	3
BP	epidermal cell differentiation	6/115	242/18481	0.00407	Tprn/Tgm1/Zfp36l1/Krt5/Krt10/Krt2	6
BP	positive regulation of miRNA transcription	3/115	52/18481	0.004156	Ctcf/Jun/Fos	3
BP	skin	7/115	326/18	0.004304	Col3a1/Tgm1/Cbx	7

	development		481		7/Zfp36l1/Krt5/Krt10/Krt2	
BP	positive regulation of protein sumoylation	2/115	16/18481	0.004351	Pias1/Pias3	2
BP	neuronal ion channel clustering	2/115	16/18481	0.004351	Ank3/Sclt1	2
BP	neuroblast division	2/115	16/18481	0.004351	Tead3/Fgf13	2
BP	keratinization	3/115	53/18481	0.004385	Tgm1/Krt5/Krt2	3
BP	positive regulation of leukocyte proliferation	5/115	175/18481	0.004774	Tlr4/Cd74/Vtcn1/Efnb1/Cd320	5
BP	cellular response to zinc ion starvation	2/115	17/18481	0.004911	Jun/Fos	2
BP	response to zinc ion starvation	2/115	17/18481	0.004911	Jun/Fos	2
BP	keratinocyte development	2/115	19/18481	0.006125	Krt10/Krt2	2
BP	contractile actin filament bundle assembly	4/115	117/18481	0.006151	Cgnl1/Phactr1/Eln/Rhpn1	4
BP	stress fiber assembly	4/115	117/18481	0.006151	Cgnl1/Phactr1/Eln/Rhpn1	4
BP	regulation of neuron migration	3/115	60/18481	0.006207	Col3a1/Arhgap32/Phactr1	3
BP	positive regulation of miRNA metabolic process	3/115	61/18481	0.006499	Ctcf/Jun/Fos	3
BP	linoleic acid metabolic process	2/115	20/18481	0.006778	Ephx2/Gstm7	2
BP	positive regulation of smooth muscle cell	2/115	20/18481	0.006778	Pias1/Olfm2	2

	differentiation					
BP	asymmetric cell division	2/115	21/18481	0.007462	Tead3/Fgf13	2
BP	response to starvation	6/115	275/18481	0.007494	Rnf167/Foxo1/Rragd/Jun/Gabarap/Fos	6
BP	positive regulation of myeloid cell differentiation	4/115	124/18481	0.00753	Cd74/Jun/Zfp36l1/Fos	4
BP	regulation of mRNA processing	4/115	125/18481	0.007743	Celf2/Rnps1/Zfp36l1/Hnrnpa2b1	4
BP	keratinocyte proliferation	3/115	67/18481	0.008421	Tgm1/Zfp36l1/Krt2	3
BP	intermediate filament organization	3/115	68/18481	0.008771	Krt5/Krt10/Krt2	3
BP	regulation of epidermis development	3/115	68/18481	0.008771	Zfp36l1/Krt10/Krt2	3
BP	chemokine (C-X-C motif) ligand 2 production	2/115	23/18481	0.008917	Tlr4/Cd74	2
BP	regulation of chemokine (C-X-C motif) ligand 2 production	2/115	23/18481	0.008917	Tlr4/Cd74	2
BP	positive regulation of non-canonical NF-kappaB signal transduction	3/115	72/18481	0.010253	Tlr4/Ank3/Cd74	3
BP	regulation of protein sumoylation	2/115	25/18481	0.010488	Pias1/Pias3	2
BP	positive regulation of membrane potential	2/115	25/18481	0.010488	Ank3/Pias3	2
BP	regulation of	3/115	73/184	0.010644	Ctcf/Jun/Fos	3

	miRNA transcription		81			
BP	ncRNA transcription	4/115	138/18481	0.010868	Ctcf/Jun/Dedd/Fos	4
BP	negative regulation of mRNA splicing, via spliceosome	2/115	26/18481	0.011317	Rnps1/Hnrnpa2b1	2
BP	myeloid cell differentiation	8/115	489/18481	0.011409	Tlr4/Thra/Cd74/Jun/Zfpm1/Zfp36l1/Pias3/Fos	8
BP	regulation of B cell proliferation	3/115	75/18481	0.011453	Tlr4/Cd74/Cd320	3
BP	miRNA transcription	3/115	75/18481	0.011453	Ctcf/Jun/Fos	3
BP	multicellular organism growth	5/115	217/18481	0.011538	Col3a1/Sp2/H3f3b/Zfp36l1/Heg1	5
BP	toll-like receptor signaling pathway	3/115	76/18481	0.011871	Tlr4/Traf3/Rab11fip2	3
BP	negative regulation of mRNA processing	2/115	27/18481	0.012173	Rnps1/Hnrnpa2b1	2
BP	response to muscle stretch	2/115	28/18481	0.013056	Jun/Fos	2
BP	regulation of B cell activation	4/115	148/18481	0.013759	Tlr4/Cd74/Zfp36l1/Cd320	4
BP	extrinsic apoptotic signaling pathway via death domain receptors	3/115	82/18481	0.014559	Gabarap/Dedd/Fgb	3
BP	muscle tissue morphogenesis	3/115	82/18481	0.014559	Col3a1/Zfpm1/Heg1	3
BP	positive regulation of epidermis development	2/115	30/18481	0.014904	Krt10/Krt2	2
BP	negative regulation of RNA splicing	2/115	31/18481	0.015868	Rnps1/Hnrnpa2b1	2
BP	outflow tract	3/115	86/184	0.016528	Jun/Zfpm1/Eln	3

	morphogenesis		81			
BP	positive regulation of macrophage cytokine production	2/115	32/18481	0.016858	Tlr4/Cd74	2
BP	positive regulation of muscle cell differentiation	3/115	87/18481	0.017042	Pias1/Thra/Olfm2	3
BP	regulation of miRNA metabolic process	3/115	87/18481	0.017042	Ctcf/Jun/Fos	3
BP	fibroblast proliferation	4/115	159/18481	0.017456	Col3a1/Sp2/Cd74/Jun	4
BP	intermediate filament cytoskeleton organization	3/115	88/18481	0.017565	Krt5/Krt10/Krt2	3
BP	positive regulation of cytokine production involved in inflammatory response	2/115	33/18481	0.017873	Tlr4/Cd74	2
BP	intermediate filament-based process	3/115	89/18481	0.018097	Krt5/Krt10/Krt2	3
BP	muscle organ morphogenesis	3/115	89/18481	0.018097	Col3a1/Zfpm1/Heg1	3
BP	mRNA processing	7/115	434/18481	0.018837	Celf2/Arvcf/Srek1ip1/Rnps1/Zfp36l1/Zcrb1/Hnrnpa2b1	7
BP	negative regulation of stress fiber assembly	2/115	34/18481	0.018914	Cgnl1/Rhpn1	2
BP	regulation of leukocyte differentiation	6/115	338/18481	0.019111	Cd74/Jun/Zfpm1/Zfp36l1/Pias3/Fos	6
BP	positive regulation of B	3/115	92/18481	0.019747	Tlr4/Cd74/Cd320	3

	cell activation					
BP	cardiac atrium morphogenesis	2/115	35/18481	0.01998	Zfpm1/Heg1	2
BP	lymphocyte costimulation	2/115	35/18481	0.01998	Efnb1/Cd320	2
BP	vitamin metabolic process	3/115	94/18481	0.020891	Aldh1l1/Rdh10/Cd320	3
BP	response to corticosteroid	6/115	349/18481	0.021969	Tlr4/Foxo1/Zfp36l1/Eln/Fos/Hnrnpa2b1	6
BP	cellular response to inorganic substance	6/115	349/18481	0.021969	Foxo1/Ank3/Jun/Eln/Fos/Krt10	6
BP	benzene-containing compound metabolic process	2/115	37/18481	0.022185	Ephx2/Gstm7	2
BP	negative regulation of Wnt signaling pathway	4/115	174/18481	0.023416	Rbms3/Foxo1/Tle1/Vgll4	4
BP	cholesterol homeostasis	3/115	99/18481	0.023909	Ephx2/Mia2/Cd320	3
BP	regulation of lymphocyte proliferation	5/115	263/18481	0.024353	Tlr4/Cd74/Vtcn1/Efnb1/Cd320	5
BP	negative regulation of myeloid cell differentiation	3/115	100/18481	0.024539	Zfpm1/Zfp36l1/Pias3	3
BP	cellular response to calcium ion	3/115	100/18481	0.024539	Jun/Fos/Krt10	3
BP	cellular component maintenance	3/115	101/18481	0.025179	Plekha7/Tprn/Adgrl3	3
BP	sterol homeostasis	3/115	101/18481	0.025179	Ephx2/Mia2/Cd320	3
BP	SMAD protein signal transduction	3/115	101/18481	0.025179	Sub1/Jun/Fos	3
BP	actin filament bundle assembly	4/115	178/18481	0.02519	Cgnl1/Phactr1/Eln/Rhpn1	4

BP	negative regulation of actin filament bundle assembly	2/115	40/18481	0.025671	Cgnl1/Rhpn1	2
BP	hippo signaling	2/115	40/18481	0.025671	Vgll4/Tead3	2
BP	cellular response to cadmium ion	2/115	40/18481	0.025671	Jun/Fos	2
BP	regulation of mononuclear cell proliferation	5/115	267/18481	0.025776	Tlr4/Cd74/Vtcn1/Efnb1/Cd320	5
BP	cellular response to metal ion	5/115	268/18481	0.026139	Ank3/Jun/Eln/Fos/Krt10	5
BP	miRNA metabolic process	3/115	103/18481	0.026484	Ctcf/Jun/Fos	3
BP	actin filament bundle organization	4/115	181/18481	0.026572	Cgnl1/Phactr1/Eln/Rhpn1	4
BP	production of molecular mediator involved in inflammatory response	3/115	104/18481	0.02715	Tlr4/Ephx2/Cd74	3
BP	regulation of mRNA splicing, via spliceosome	3/115	104/18481	0.02715	Celf2/Rnps1/Hnrnpa2b1	3
BP	lung development	5/115	273/18481	0.028008	Col3a1/Thra/Rdh10/Heg1/Hnrnpa2b1	5
BP	cellular response to oxygen levels	5/115	274/18481	0.028392	Foxo1/Jun/Zfp36l1/Eln/Fos	5
BP	positive regulation of ubiquitin-dependent protein catabolic process	3/115	106/18481	0.02851	Pias1/Agtpbp1/Gabarap	3
BP	cellular response to tumor necrosis factor	5/115	275/18481	0.028779	Traf3/Zfp36l1/Pias3/Ybx3/Fos	5
BP	respiratory tube development	5/115	276/18481	0.029169	Col3a1/Thra/Rdh10/Heg1/Hnrnpa2b1	5
BP	cardiac atrium	2/115	43/184	0.029364	Zfpm1/Heg1	2

	development		81			
BP	regulation of smooth muscle cell differentiation	2/115	43/184 81	0.029364	Pias1/Olfm2	2
BP	muscle organ development	6/115	375/18 481	0.02984	Col3a1/Zfpm1/He g1/Eln/Ybx3/Fos	6
BP	positive regulation of myeloid leukocyte cytokine production involved in immune response	2/115	44/184 81	0.03064	Tlr4/Cd74	2
BP	protein polymerization	5/115	280/18 481	0.030764	Fgf13/Eln/Vtn/Krt 5/Fgb	5
BP	B cell proliferation	3/115	110/18 481	0.031334	Tlr4/Cd74/Cd320	3
BP	epidermis development	6/115	381/18 481	0.031889	Tprn/Tgm1/Zfp36l 1/Krt5/Krt10/Krt2	6
BP	response to calcium ion	4/115	193/18 481	0.032551	Jun/Fos/Fgb/Krt10	4
BP	positive regulation of leukocyte differentiation	4/115	193/18 481	0.032551	Cd74/Jun/Zfp36l1/ Fos	4
BP	positive regulation of hemopoiesis	4/115	193/18 481	0.032551	Cd74/Jun/Zfp36l1/ Fos	4
BP	regulation of chemokine production	3/115	112/18 481	0.0328	Tlr4/Cd74/Zfpm1	3
BP	chemokine production	3/115	114/18 481	0.034301	Tlr4/Cd74/Zfpm1	3
BP	regulation of leukocyte proliferation	5/115	289/18 481	0.03455	Tlr4/Cd74/Vtcn1/ Efnb1/Cd320	5
BP	microglial cell activation	2/115	47/184 81	0.034595	Tlr4/Jun	2
BP	macrophage cytokine production	2/115	47/184 81	0.034595	Tlr4/Cd74	2

BP	regulation of macrophage cytokine production	2/115	47/18481	0.034595	Tlr4/Cd74	2
BP	regulation of non-canonical NF-kappaB signal transduction	3/115	116/18481	0.035837	Tlr4/Ank3/Cd74	3
BP	protein sumoylation	2/115	48/18481	0.035955	Pias1/Pias3	2
BP	extracellular matrix assembly	2/115	49/18481	0.037335	Col3a1/Eln	2
BP	RNA splicing	6/115	396/18481	0.037409	Celf2/Arvcf/Srek1ip1/Rnps1/Zcrb1/Hnrnpa2b1	6
BP	learning	4/115	203/18481	0.038089	Pias1/Jun/Fgf13/Fos	4
BP	positive regulation of protein modification by small protein conjugation or removal	3/115	120/18481	0.039014	Pias1/Gabarap/Pias3	3
BP	leukocyte activation involved in inflammatory response	2/115	51/18481	0.040156	Tlr4/Jun	2
BP	positive regulation of cell-cell adhesion	5/115	302/18481	0.040512	Ank3/Cd74/Vtcn1/Efnb1/Fgb	5
BP	response to tumor necrosis factor	5/115	302/18481	0.040512	Traf3/Zfp36l1/Pias3/Ybx3/Fos	5
BP	negative regulation of leukocyte differentiation	3/115	122/18481	0.040655	Cd74/Zfpm1/Pias3	3
BP	toll-like receptor 4 signaling pathway	2/115	52/18481	0.041597	Tlr4/Rab11fip2	2

BP	positive regulation of TORC1 signaling	2/115	52/18481	0.041597	Rnf167/Rragd	2
BP	positive regulation of protein catabolic process	4/115	210/18481	0.042269	Foxo1/Pias1/Vgll4/Gabarap	4
BP	anterograde axonal transport	2/115	53/18481	0.043056	Ank3/Agtpbp1	2
BP	regulation of keratinocyte proliferation	2/115	53/18481	0.043056	Tgm1/Zfp36l1	2
BP	response to hyperoxia	2/115	53/18481	0.043056	Foxo1/Eln	2
BP	cellular response to transforming growth factor beta stimulus	5/115	308/18481	0.043462	Col3a1/Jun/Zfp36l1/Eln/Fos	5
BP	respiratory system development	5/115	309/18481	0.043966	Col3a1/Thra/Rdh10/Heg1/Hnrnpa2b1	5
BP	cellular response to glucocorticoid stimulus	3/115	126/18481	0.04404	Foxo1/Zfp36l1/Eln	3
BP	response to glucocorticoid	5/115	311/18481	0.044984	Tlr4/Foxo1/Zfp36l1/Eln/Fos	5
BP	cilium organization	6/115	415/18481	0.045234	Cep350/Rabep2/Dusp23/Ift70a2/Dclk1/Sclt1	6
BP	positive regulation of lymphocyte activation	5/115	312/18481	0.045499	Tlr4/Cd74/Vtcn1/Efnb1/Cd320	5
BP	cerebral cortex cell migration	2/115	55/18481	0.046031	Col3a1/Fgf13	2
BP	actomyosin structure organization	4/115	216/18481	0.046051	Cgnl1/Phactr1/Eln/Rhpn1	4
BP	cellular response to amino acid starvation	2/115	56/18481	0.047546	Rnf167/Rragd	2
BP	response to immobilization	2/115	56/18481	0.047546	Ank3/Fos	2

	stress					
BP	negative regulation of hemopoiesis	3/115	130/18481	0.047561	Cd74/Zfpm1/Pias3	3
BP	response to transforming growth factor beta	5/115	316/18481	0.047592	Col3a1/Jun/Zfp36l1/Eln/Fos	5
BP	adaptive immune response	6/115	423/18481	0.048814	Tlr4/Ebag9/Cd74/Vtcn1/C4a/Fgb	6
BP	regulation of erythrocyte differentiation	2/115	57/18481	0.049079	Zfpm1/Zfp36l1	2
CC	transcription regulator complex	11/123	500/18710	0.000464	Taf4/Sub1/Tle1/E2f3/Taf3/Thra/Tead3/Jun/Zfpm1/Taf1a/Fos	11
CC	RNA polymerase II transcription regulator complex	7/123	235/18710	0.000933	Taf4/Tle1/E2f3/Taf3/Thra/Jun/Fos	7
CC	keratin filament	4/123	85/18710	0.002389	Krt6a/Krt5/Krt10/Krt2	4
CC	adherens junction	5/123	157/18710	0.003812	Shroom3/Plekha7/Pdlim2/Arvcf/Tgm1	5
CC	cornified envelope	3/123	52/18710	0.004846	Krt5/Krt10/Krt2	3
CC	U12-type spliceosomal complex	2/123	28/18710	0.014493	Snrnp48/Zcrb1	2
CC	phagocytic cup	2/123	30/18710	0.016536	Tlr4/Rab11fip2	2
CC	male germ cell nucleus	3/123	82/18710	0.016848	Ctcf/Taf4/Taf3	3
CC	apical junction complex	4/123	150/18710	0.017278	Shroom3/Plekha7/Ank3/Cgnl1	4
CC	transcription factor TFIID complex	2/123	33/18710	0.019817	Taf4/Taf3	2
CC	intermediate filament	4/123	163/18710	0.022663	Krt6a/Krt5/Krt10/Krt2	4

CC	germ cell nucleus	3/123	109/18710	0.035189	Ctcf/Taf4/Taf3	3
CC	neuron projection cytoplasm	3/123	111/18710	0.036835	Ank3/Agtpbp1/Hnrnpa2b1	3
CC	chromosome, telomeric region	3/123	113/18710	0.03852	H3f3b/Tox4/Hnrnpa2b1	3
CC	plasma membrane bounded cell projection cytoplasm	5/123	282/18710	0.038639	Ank3/Agtpbp1/Ift70a2/Gabarap/Hnrnpa2b1	5
CC	spliceosomal complex	4/123	199/18710	0.04245	Snrnp48/Dqx1/Zcrb1/Hnrnpa2b1	4
CC	intermediate filament cytoskeleton	4/123	199/18710	0.04245	Krt6a/Krt5/Krt10/Krt2	4
CC	chloride channel complex	2/123	51/18710	0.044343	Ttyh3/Clic3	2
MF	structural constituent of skin epidermis	3/110	29/17065	0.000843	Krt5/Krt10/Krt2	3
MF	chromatin DNA binding	5/110	119/17065	0.001036	Ctcf/Foxo1/H3f3b/Thra/Tox4	5
MF	microtubule binding	7/110	273/17065	0.001947	Eml3/Cep350/MAST1/Dclk1/Mx1/Fgf13/Gabarap	7
MF	SUMO ligase activity	2/110	12/17065	0.002605	Pias1/Pias3	2
MF	tubulin binding	8/110	388/17065	0.00363	Eml3/Cep350/MAST1/Agtpbp1/Dclk1/Mx1/Fgf13/Gabarap	8
MF	core promoter sequence-specific DNA binding	3/110	48/17065	0.003651	Taf4/H3f3b/Fos	3
MF	mRNA 3'-UTR binding	6/110	231/17065	0.003835	Celf2/Rbms3/Rnps1/Zfp36l1/Ybx3/Hnrnpa2b1	6
MF	protein heterodimerization activity	7/110	339/17065	0.006376	Tlr4/Taf4/Taf3/H3f3b/Rragd/H2aj/Krt10	7
MF	cargo receptor	3/110	62/170	0.00748	Mia2/Vtn/Cd320	3

	activity		65			
MF	RNA polymerase II core promoter sequence-specific DNA binding	2/110	22/17065	0.008744	H3f3b/Fos	2
MF	RNA polymerase II-specific DNA-binding transcription factor binding	7/110	381/17065	0.011689	Taf4/Ank3/Tead3/Jun/Zfpm1/Pias3/Fos	7
MF	R-SMAD binding	2/110	26/17065	0.012097	Jun/Fos	2
MF	transcription coregulator binding	4/110	139/17065	0.012518	Ctcf/Foxo1/Vgll4/Fos	4
MF	SUMO transferase activity	2/110	27/17065	0.013011	Pias1/Pias3	2
MF	mRNA 3'-UTR AU-rich region binding	2/110	28/17065	0.013953	Rbms3/Zfp36l1	2
MF	ubiquitin protein ligase binding	6/110	318/17065	0.017008	Foxo1/Pias1/Traf3/Pdlim2/Jun/Gabarap	6
MF	SMAD binding	3/110	84/17065	0.017023	Col3a1/Jun/Fos	3
MF	hydrolase activity, hydrolyzing N-glycosyl compounds	2/110	33/17065	0.019088	Tlr4/Macrod2	2
MF	sodium channel regulator activity	2/110	35/17065	0.021332	Fgf13/Sclt1	2
MF	ubiquitin-like protein ligase binding	6/110	336/17065	0.021645	Foxo1/Pias1/Traf3/Pdlim2/Jun/Gabarap	6
MF	single-stranded RNA binding	3/110	97/17065	0.024812	Rbms3/Thra/Cbx7	3
MF	pre-mRNA binding	2/110	40/17065	0.027389	Celf2/Hnrnpa2b1	2
MF	beta-tubulin	2/110	45/170	0.03405	Fgf13/Gabarap	2

	binding		65			
MF	cell adhesion molecule binding	5/110	290/17065	0.039669	Ank3/Col3a1/Arvcf/Vtn/Fgb	5
MF	single-stranded DNA binding	3/110	122/17065	0.044322	Sub1/Ybx3/Hnrnpa2b1	3
MF	DNA-binding transcription repressor activity, RNA polymerase II-specific	5/110	300/17065	0.044778	Ctcf/Foxo1/Sp2/Jun/Zbtb2	5
MF	DNA-binding transcription repressor activity	5/110	306/17065	0.048025	Ctcf/Foxo1/Sp2/Jun/Zbtb2	5
MF	transcription coactivator binding	2/110	55/17065	0.04901	Foxo1/Vgll4	2
