# Supplementary material for: The Altered Proteomic Landscape in Renal Tubular Epithelial Cells under High Oxalate Stimulation
Source: Biology (Basel). 2024 Oct 11;13(10):814. doi: 10.3390/biology13100814 (PMC11505525; doi:10.3390/biology13100814)
Supplement: Supplementary file 1 [file biology-13-00814-s001.zip › Table S4.pdf]

Table S4. The detailed results of KEGG enrichment analysis of the up-regulated DEPs.

Description	Protein Ratio	Bg Ratio	P-value	Protein ID	Count
Amoebiasis	5/68	99/10049	0.000535	Tgfb2/Lamb3/Il1r1/Lama3/Cd14	5
Cell cycle	6/68	159/10049	0.000696	Pds5b/Anapc15/Cdc14a/Sfn/Tgfb2/Cdkn2d	6
Toxoplasmosis	5/68	114/10049	0.001015	RT1-DMb/Tgfb2/Ifngr1/Lamb3/Lama3	5
Leishmaniasis	4/68	74/10049	0.001546	RT1-DMb/Ptgs2/Tgfb2/Ifngr1	4
Complement and coagulation cascades	4/68	87/10049	0.0028	F9/F5/Serpine1/F3	4
Ferroptosis	3/68	48/10049	0.004124	Steap3/Map1lc3b/Slc39a14	3
NF-kappa B signaling pathway	4/68	100/10049	0.00462	Ptgs2/Tirap/Il1r1/Cd14	4
Ovarian steroidogenesis	3/68	58/10049	0.007012	Ptgs2/Hsd17b7/Igf1r	3
Inflammatory bowel disease	3/68	61/10049	0.008062	RT1-DMb/Tgfb2/Ifngr1	3
Tuberculosis	5/68	189/10049	0.008938	RT1-DMb/Tirap/Tgfb2/Ifngr1/Cd14	5
MAPK signaling pathway	6/68	302/10049	0.016123	Daxx/Epha2/Tgfb2/Il1r1/Igf1r/Cd14	6
p53 signaling pathway	3/68	81/10049	0.01737	Steap3/Sfn/Serpine1	3
ECM-receptor interaction	3/68	90/10049	0.022914	Spp1/Lamb3/Lama3	3
HIF-1 signaling pathway	4/68	161/10049	0.023398	Serpine1/Timp1/Ifngr1/Igf1r	4
Efferocytosis	4/68	162/10049	0.023871	Ptgs2/Mfge8/Mertk/Axl	4
Hematopoietic cell lineage	3/68	92/10049	0.024263	RT1-DMb/Il1r1/Cd14	3
Fructose and mannose metabolism	2/68	36/10049	0.024515	Tigar/Pfkfb4	2
Small cell lung cancer	3/68	101/10049	0.030852	Ptgs2/Lamb3/Lama3	3
Cell adhesion	4/68	178/10	0.032239	Nfasc/RT1-	4

molecules		049		DMb/Ntng2/Sdc2	
Toll-like receptor signaling pathway	3/68	103/10049	0.032432	Tirap/Spp1/Cd14	3
Th17 cell differentiation	3/68	104/10049	0.033237	RT1-DMb/Ifngr1/Il1r1	3
AGE-RAGE signaling pathway in diabetic complications	3/68	104/10049	0.033237	Serpine1/Tgfb2/F3	3
Chagas disease	3/68	106/10049	0.03488	Serpine1/Tgfb2/Ifngr1	3
Cytokine-cytokine receptor interaction	5/68	274/10049	0.037622	Il13ra1/Tnfrsf12a/Tgfb2/Ifngr1/Il1r1	5
Transcriptional misregulation in cancer	4/68	193/10049	0.041473	Bmp2k/Igf1r/Arnt2/Cd14	4
TNF signaling pathway	3/68	119/10049	0.04656	Ptgs2/Ifi47/Ripk3	3
Focal adhesion	4/68	203/10049	0.048384	Spp1/Lamb3/Igf1r/Lama3	4
