# Supplementary material for: The Altered Proteomic Landscape in Renal Tubular Epithelial Cells under High Oxalate Stimulation
Source: Biology (Basel). 2024 Oct 11;13(10):814. doi: 10.3390/biology13100814 (PMC11505525; doi:10.3390/biology13100814)
Supplement: Supplementary file 1 [file biology-13-00814-s001.zip › Table S5.pdf]

Table S5. The detailed results of KEGG enrichment analysis of the down-regulated DEPs.

Description	Protein Ratio	Bg Ratio	P-value	Protein ID	Count
Pertussis	4/53	74/10049	0.000604	Tlr4/Jun/C4a/Fos	4
Measles	5/53	155/10049	0.001293	Tlr4/Traf3/Jun/Mx1/Fos	5
Hepatitis B	5/53	169/10049	0.001896	Tlr4/Traf3/E2f3/Jun/Fos	5
Toll-like receptor signaling pathway	4/53	103/10049	0.002074	Tlr4/Traf3/Jun/Fos	4
Estrogen signaling pathway	4/53	138/10049	0.005924	Ebag9/Jun/Fos/Krt10	4
Alcoholic liver disease	4/53	140/10049	0.006231	Tlr4/Foxo1/Traf3/C4a	4
Leishmaniasis	3/53	74/10049	0.006898	Tlr4/Jun/Fos	3
Complement and coagulation cascades	3/53	87/10049	0.010752	C4a/Vtn/Fgb	3
Rheumatoid arthritis	3/53	87/10049	0.010752	Tlr4/Jun/Fos	3
PD-L1 expression and PD-1 checkpoint pathway in cancer	3/53	90/10049	0.011786	Tlr4/Jun/Fos	3
IL-17 signaling pathway	3/53	91/10049	0.012143	Traf3/Jun/Fos	3
Hepatitis C	4/53	172/10049	0.012651	Pias1/Traf3/E2f3/Mx1	4
Chemical carcinogenesis - reactive oxygen species	5/53	266/10049	0.012756	Ephx2/Gstm7/Jun/Cox6c/Fos	5
Endocrine resistance	3/53	97/10049	0.014415	E2f3/Jun/Fos	3
Neutrophil extracellular trap formation	4/53	185/10049	0.016147	Tlr4/H3f3b/H2aj/Fgb	4
NOD-like receptor signaling pathway	4/53	186/10049	0.016438	Tlr4/Traf3/Jun/Gabarap	4
AGE-RAGE signaling pathway in diabetic complications	3/53	104/10049	0.017353	Foxo1/Col3a1/Jun	3
Chagas disease	3/53	106/10049	0.018249	Tlr4/Jun/Fos	3
Basal transcription factors	2/53	44/10049	0.022418	Taf4/Taf3	2
Systemic lupus	3/53	115/10	0.022598	H3f3b/C4a/H2aj	3

erythematosus		049			
TNF signaling pathway	3/53	119/10049	0.024697	Traf3/Jun/Fos	3
Lipid and atherosclerosis	4/53	223/10049	0.029636	Tlr4/Traf3/Jun/Fos	4
Relaxin signaling pathway	3/53	129/10049	0.030392	Col3a1/Jun/Fos	3
Kaposi sarcoma-associated herpesvirus infection	4/53	225/10049	0.030487	Traf3/E2f3/Jun/Fos	4
Cytoskeleton in muscle cells	4/53	231/10049	0.033127	Ank3/Pdlim2/Col3a1/Eln	4
Chemical carcinogenesis - receptor activation	4/53	234/10049	0.034496	Ephx2/Gstm7/Jun/Fos	4
Yersinia infection	3/53	139/10049	0.036722	Tlr4/Jun/Fos	3
Apoptosis	3/53	141/10049	0.038064	Jun/Ctso/Fos	3
Inflammatory bowel disease	2/53	61/10049	0.04098	Tlr4/Jun	2
Breast cancer	3/53	151/10049	0.045145	E2f3/Jun/Fos	3
Fluid shear stress and atherosclerosis	3/53	154/10049	0.047389	Gstm7/Jun/Fos	3
Amphetamine addiction	2/53	68/10049	0.049843	Jun/Fos	2
