# Supplementary material for: The Altered Proteomic Landscape in Renal Tubular Epithelial Cells under High Oxalate Stimulation
Source: Biology (Basel). 2024 Oct 11;13(10):814. doi: 10.3390/biology13100814 (PMC11505525; doi:10.3390/biology13100814)
Supplement: Supplementary file 1 [file biology-13-00814-s001.zip › Table S6.pdf]

Table S6. The specific PPI information of DEPs.

Protein 1	Protein 2	Combined score
AASDH	EPHX2	432
AASDH	ALDH1L1	700
AASDH	LOC684270	419
ADAT1	ALDH1L1	400
ADGRG1	TSPAN3	729
ANK3	ANKS1A	459
ANK3	NFASC	986
APOH	TLR4	743
APOH	F3	531
APOH	LRP8	722
APOH	ITIH2	763
APOH	FGB	845
ARHGAP32	GABARAP	504
ARNT2	PER1	580
ARVCF	PLEKHA7	828
AXL	MFGE8	556
BCL7A	MLLT10	455
BRCA2	PDS5B	960
BRCA2	RAD18	445
C4	VTN	550
C4	VTN	540
CBX7	ANAPC15	722
CD14	TIRAP	737
CD74	RT1-DMB	986
CDC14A	FZD7	598
CELF2	MLLT10	489
CEP350	SCLT1	457
CEP350	TPCN2	630
CGNL1	PLEKHA7	516
CLIC3	IER3IP1	447
CLIC3	RBMS3	420
COL3A1	TIMP1	713
COL3A1	LAMA3	470
COL3A1	ADGRG1	536
COL3A1	ELN	700
CTCF	TAF3	595
CTCF	ZNF513	511
CTCF	PDS5B	410
CYB5B	ALAS1	593
DAXX	H3-3B	745
DAXX	RIPK3	613
DAXX	PIAS1	446

DHRS9	RETSAT	901
DUSP23	FZD7	477
E2F3	FZD7	514
EFNB1	EPHA2	954
ELN	VTN	730
ELN	MFGE8	648
ELN	SPP1	629
EPHA2	ARVCF	634
EPHA2	ANKS1A	405
EPHX2	HAGHL	408
EPHX2	GLRX2	459
EPHX2	ALDH1L1	492
EPHX2	LOC684270	491
ERG28	IER3IP1	487
F3	F5	789
F3	VTN	403
F5	ITIH2	473
F9	APOH	403
F9	F3	978
F9	F5	600
F9	ITIH2	584
F9	VTN	563
F9	FGB	717
FAM193B	PHACTR1	623
FOS	TRAF3	543
FOS	JUN	999
FOS	TIMP1	436
FOS	TLR4	612
FOS	FOXO1	461
FOS	IGF1R	483
FOS	CTCF	422
FOS	SPP1	478
FOXO1	IGF1R	711
FOXO1	SFN	972
FOXO1	FZD7	522
GABARAP	ANK3	849
GABARAP	MAP1LC3B	924
GPNMB	SPP1	405
H3-3B	CBX7	493
H3-3B	TAF3	405
HNRNPA2B1	RBMS3	419
HSD17B7	ERG28	971
HSD17B7	CYP4V2	555
IFI47	MX1	440

IFNGR1	RIPK3	428
IGF1R	SFN	470
IGF1R	ARVCF	526
IL13RA1	TENT5D	652
IL1R1	RIPK3	442
IL1R1	TIRAP	497
ITIH2	TTC30A2	622
ITIH2	VTN	498
ITIH2	FGB	726
JUN	TIMP1	463
JUN	TLR4	657
JUN	ARNT2	675
JUN	FOXO1	577
JUN	IGF1R	486
JUN	IL1R1	525
JUN	CTCF	435
JUN	GSTM7	605
JUN	TIRAP	437
JUN	SPP1	410
KCTD7	DCUN1D2	600
KRT10	KRT2	934
KRT5	KRT10	937
KRT5	KRT2	851
LAMA3	ARHGAP29	506
LAMB3	KRT5	434
LAMB3	LAMA3	885
LAMB3	LPGAT1	407
LRP12	SDC2	900
LRP12	ST7	400
MAGEH1	PDS5B	406
MAGEH1	GSPT2	425
MAST1	DUSP23	589
MAST1	CDC14A	589
MCOLN1	TPCN2	808
MERTK	CD14	401
MERTK	AXL	575
MERTK	MFGE8	746
MIA2	VTCN1	495
MLLT10	ZNF260	423
MLLT10	ETL4	402
MTHFD2	SSNA1	474
MTHFD2	SLC19A1	548
MTHFD2	ALDH1L1	402
NAP1L1	VGLL4	474

PDLIM2	IGF1R	440
PDLIM2	MICALL1	660
PDP2	TOX4	750
PDP2	PDHA1L1	951
PDP2	TIGAR	469
PFKFB4	TIGAR	950
PHACTR1	ETL4	463
PIAS1	RAD18	641
PIAS3	RAD18	641
PIGB	AGTPBP1	491
PLEKHH1	RBPMS2	527
PSMC3	PDHA1L1	404
PTGS2	FOS	690
PTGS2	JUN	677
PTGS2	TIMP1	586
PTGS2	TLR4	792
PTGS2	F3	490
PTGS2	IGF1R	453
PTGS2	IL1R1	486
PTGS2	SPP1	538
PTGS2	OTULINL	511
RAB11FIP2	MICALL1	481
RBMX	HNRNPA2B1	476
RDH10	RETSAT	918
RHBDL3	MFGE8	509
RHPN1	OLFM2	578
RIPK3	TIRAP	409
SAP30	TAF3	514
SAP30	IFRD1	411
SCLT1	PDS5B	521
SDC2	LRP8	900
SDC2	VTN	631
SERPINE1	TGFB2	469
SERPINE1	PTGS2	609
SERPINE1	F9	533
SERPINE1	TNFRSF12A	576
SERPINE1	COL3A1	508
SERPINE1	FOS	458
SERPINE1	JUN	498
SERPINE1	TIMP1	762
SERPINE1	TLR4	571
SERPINE1	F3	819
SERPINE1	LRP8	620
SERPINE1	ELN	527

SERPINE1	VTN	999
SERPINE1	FGB	419
SERPINE1	SPP1	590
SFN	FZD7	509
SLC25A36L1	ZNF260	507
SLC29A3	ADGRE5	562
SLC29A3	MFSD9	663
SLC39A14	STEAP3	665
SLC39A14	ALAS1	421
SNX10	ZCCHC9	511
SPSB1	DCUN1D2	607
STEAP3	ALAS1	420
SUPT5H	BMP2K	492
SUPT5H	SUB1	727
TAF3	TAF4	959
TAF4	DMWD	404
TANK	TLR4	749
TANK	CD14	607
TANK	RIPK3	480
TCEANC	SUPT5H	684
TEAD3	VGLL4	758
TEAD3	FOS	423
TGFB2	COL3A1	561
TGFB2	FOS	708
TGFB2	JUN	729
TGFB2	TIMP1	448
TGFB2	FGF13	414
TGM1	KRT10	700
TGM1	KRT2	557
THRA	ARNT2	469
TIMP1	TLR4	508
TIMP1	TSPAN3	722
TIMP1	CD14	469
TIMP1	ELN	723
TIMP1	VTN	706
TIMP1	SPP1	698
TLR4	F3	525
TLR4	IFNGR1	443
TLR4	FOXO1	466
TLR4	IL1R1	774
TLR4	MERTK	404
TLR4	CD14	999
TLR4	CD74	488
TLR4	RIPK3	636

TLR4	ELN	470
TLR4	TIRAP	999
TLR4	MX1	447
TLR4	SPP1	454
TNFRSF12A	TRAF3	798
TNFRSF12A	TIMP1	580
TNFRSF12A	FGF13	457
TPRN	CLIC3	507
TRAF3	JUN	402
TRAF3	TANK	998
TRAF3	TLR4	966
TRAF3	IL1R1	578
TRAF3	CD14	746
TRAF3	RIPK3	433
TRAF3	TIRAP	761
TSPAN3	MFGE8	405
TTLL4	AGTPBP1	421
TTLL4	PHACTR1	478
TTLL4	ETL4	405
USP2	RHPN1	410
USP2	OTUD3	695
USP2	PER1	806
VTN	FGB	731
VTN	SPP1	768
ZCRB1	HNRNPA2B1	452
ZCRB1	SNRNP48	872
ZNF260	GSPT2	530
ZSCAN21	CTCF	507
ZSCAN21	ZNF513	510
