# Supplementary material for: The Altered Proteomic Landscape in Renal Tubular Epithelial Cells under High Oxalate Stimulation
Source: Biology (Basel). 2024 Oct 11;13(10):814. doi: 10.3390/biology13100814 (PMC11505525; doi:10.3390/biology13100814)
Supplement: Supplementary file 1 [file biology-13-00814-s001.zip › Table S7.pdf]

Table S7. The expression levels of DEPs in oxalate-treated cells from our proteomics data and hyperoxaluric stone forming rats from the public dataset.

All DEPs identified in our study	Our proteomics data		The public proteomics data	
	Fold change (oxalate-treated cells versus untreated cells)	P-value	Fold change (hyperoxaluric stone forming rats versus normal rats)	P-value
Ggt1	1.92016	8.94E-06	0.7114	0.046013
Epha2	1.755082	9.45E-06	Not available	Not available
Spp1	1.700031	3.1E-05	5.6841	4.34E-05
Lamb3	1.544646	4.61E-05	Not available	Not available
F5	1.916783	5.74E-05	Not available	Not available
Slc19a1	1.988778	5.81E-05	0.473	0.061035
Arvcf	0.604323	7.03E-05	0.7797	0.032887
Plekha7	0.650976	7.08E-05	0.817	0.067706
C8h15orf39	0.628729	0.000123	Not available	Not available
Axl	1.6003	0.000129	Not available	Not available
F3	1.531681	0.000139	Not available	Not available
Ripk3	1.666865	0.000154	Not available	Not available
Arhgap29	0.659065	0.000161	Not available	Not available
Tspan3	1.534508	0.000189	Not available	Not available
Dhrs9	1.530635	0.000232	Not available	Not available
Cd14	1.511262	0.000238	1.6287	0.063328
Dnaaf2	1.553062	0.000307	Not available	Not available
Sub1	0.630874	0.000319	0.1932	0.001961
Sdhaf2	4.339108	0.000321	1.2896	0.65116
Sfn	1.814227	0.000348	1.814	0.123213
Mfge8	1.789026	0.000356	1.5066	0.01754
Tgm1	0.575228	0.000387	Not available	Not available
Bbs9	1.592622	0.000388	Not available	Not available
Rnps1	0.563762	0.000391	0.2917	4.9E-05
Bmp2k	1.535747	0.000399	Not available	Not available
Mthfd2	1.568978	0.000412	Not available	Not available
Heg1	0.443712	0.000466	Not available	Not available
Pex2	1.50516	0.000475	Not available	Not available
Ptgs2	1.949453	0.000516	Not available	Not available
Shroom3	0.657274	0.000546	0.7491	0.359167
Dclk1	0.579444	0.000568	1.307	0.78834
F9	4.063229	0.000591	0.9911	0.982389
Lpgat1	1.568595	0.00063	0.8487	0.222021
Hsd17b7	1.662746	0.000682	Not available	Not available
Ttyh3	0.653595	0.000727	Not available	Not available
LOC684270	0.633805	0.000738	0.7148	0.072431
Hnrnpk-ps1	1.955431	0.000925	Not available	Not available

Taf4	0.653897	0.000938	Not available	Not available
Glrx2	1.879336	0.000945	Not available	Not available
Cgnl1	0.487103	0.00103	0.7695	0.049455
Cyb5b	1.569526	0.001041	0.8792	0.378897
Arnt2	1.523922	0.001111	Not available	Not available
Mier3	1.836745	0.00115	Not available	Not available
Steap3	1.918301	0.001203	Not available	Not available
Trak2	1.703802	0.001212	Not available	Not available
Serpine1	1.735992	0.001291	Not available	Not available
Selenbp1	0.607563	0.001294	Not available	Not available
Crmp1	1.616745	0.00133	Not available	Not available
Cdk5rap1	1.622673	0.001336	Not available	Not available
Tle1	0.614934	0.001415	Not available	Not available
Cd320	0.24065	0.001416	Not available	Not available
Lama3	1.51945	0.001486	Not available	Not available
Fastkd3	0.44204	0.001608	Not available	Not available
Snrnp48	0.649558	0.001632	Not available	Not available
Supt5h	4.845684	0.001744	1.0365	0.665522
Map1lc3b	1.850417	0.001835	Not available	Not available
Rab11fip2	0.597686	0.001912	Not available	Not available
Pigb	1.542278	0.001915	Not available	Not available
Zc3hav1l	0.620526	0.001936	Not available	Not available
Fgb	0.179766	0.001976	1.5481	0.068324
Eln	0.443442	0.002044	1.2159	0.984036
Pdp2	1.842622	0.002064	0.9321	0.643161
Ephx2	0.659453	0.002102	0.7615	0.190954
Tgfb2	1.726471	0.002157	Not available	Not available
RGD1304884	1.576858	0.002189	Not available	Not available
Rabep2	0.634228	0.002193	0.9656	0.537908
Mmtag2	0.580702	0.002265	Not available	Not available
Brca2	1.860282	0.002267	Not available	Not available
Akap2	0.639326	0.002285	0.9214	0.477138
Tram1	3.331524	0.002322	0.9497	0.92665
Tank	1.718373	0.002376	Not available	Not available
Timp1	1.725412	0.002489	Not available	Not available
Anapc15	2.555726	0.002552	Not available	Not available
Anxa10	1.927259	0.002558	Not available	Not available
Rad18	2.034317	0.002573	Not available	Not available
Cracdl	1.632645	0.002654	Not available	Not available
Gstm7	0.621223	0.002752	Not available	Not available
Pias1	0.641775	0.002762	Not available	Not available
Fos	0.213239	0.002926	Not available	Not available
Erg28	1.567799	0.002927	Not available	Not available
Rbpms2	1.610673	0.00295	Not available	Not available

Celf2	0.66222	0.002969	1.315	0.157318
Krt2	0.091242	0.002975	Not available	Not available
Fam193b	0.63859	0.003069	Not available	Not available
Igf1r	1.524232	0.003251	Not available	Not available
Foxo1	0.649315	0.003434	Not available	Not available
Krt10	0.101457	0.003522	Not available	Not available
Gabarap	0.49671	0.003798	Not available	Not available
Ier3ip1	1.808554	0.00405	Not available	Not available
Mllt10	0.645221	0.004097	Not available	Not available
Ctcf	0.666923	0.004114	Not available	Not available
Zfp36l1	0.460735	0.004216	Not available	Not available
RGD1565222	0.434657	0.004771	Not available	Not available
Ttpa	1.807555	0.004802	Not available	Not available
Mertk	1.776821	0.004898	Not available	Not available
Anks1a	2.053842	0.004953	2.2697	0.002059
Uvssa	0.611312	0.004996	Not available	Not available
Ank3	0.648107	0.005124	Not available	Not available
Tmem129	1.889432	0.005191	Not available	Not available
Pfkfb4	1.552326	0.005448	Not available	Not available
Atl1	0.448305	0.005578	Not available	Not available
Zcrb1	0.378696	0.006078	Not available	Not available
Tpcn2	2.024836	0.006169	Not available	Not available
E2f3	0.611504	0.006194	Not available	Not available
Ebag9	0.643906	0.006203	Not available	Not available
Vgll4	0.613728	0.006436	Not available	Not available
Vtn	0.266254	0.006505	1.1996	0.339499
Aldh1l1	0.566372	0.006961	0.9846	0.821244
Fbxl16	0.584844	0.007013	Not available	Not available
Sclt1	0.385151	0.0071	Not available	Not available
Alas1	1.750743	0.007103	Not available	Not available
Rnf167	0.654574	0.007232	Not available	Not available
Snx10	1.751291	0.007307	Not available	Not available
Ybx3	0.236599	0.007331	1.6465	0.00184
Sap30	2.306087	0.007375	Not available	Not available
Tmem135	1.532279	0.007434	1.0042	0.785839
Pdha1l1	2.24391	0.007664	Not available	Not available
Tox4	0.54984	0.007889	Not available	Not available
Micall1	1.763505	0.007929	Not available	Not available
Tcp11l2	0.540924	0.008098	Not available	Not available
Adgrg1	2.497575	0.008322	Not available	Not available
Mfsd9	1.530668	0.008358	Not available	Not available
Dqx1	0.568755	0.008391	Not available	Not available
Szrd1	0.624128	0.008705	Not available	Not available
Taf3	0.608551	0.008736	Not available	Not available

Mast1	0.622928	0.008764	Not available	Not available
Ttc30a2	0.58293	0.009096	Not available	Not available
RT1-DMb	2.209451	0.009499	Not available	Not available
Gas2l3	2.065585	0.009522	Not available	Not available
Arhgap32	0.520726	0.009544	Not available	Not available
Krt6a	0.261674	0.009673	Not available	Not available
Tnfaip8l1	1.568938	0.009759	Not available	Not available
Ppfibp2	0.666165	0.009768	Not available	Not available
Cox6c2	0.483143	0.009953	Not available	Not available
Adat1	1.863924	0.010084	Not available	Not available
Aasdh	1.524248	0.010125	Not available	Not available
Col3a1	0.619997	0.010292	1.613	0.090547
Itih2	0.39095	0.010301	1.3157	0.194119
Adgrl3	0.571416	0.010666	Not available	Not available
Fam83d	1.517356	0.01089	Not available	Not available
Elk3	1.658674	0.010923	Not available	Not available
Pxylp1	0.574341	0.011033	Not available	Not available
Prr15l	0.637618	0.01107	Not available	Not available
Sp2	0.602481	0.011283	Not available	Not available
Tigar	1.604685	0.011442	Not available	Not available
Pias3	0.450772	0.011795	Not available	Not available
Mia2	0.581198	0.011829	0.9384	0.410111
Cdkn2d	1.724812	0.011932	Not available	Not available
Psmc3	2.260189	0.011952	1.0654	0.14181
Zfand2a	2.722962	0.012487	Not available	Not available
Hirip3	0.665926	0.012669	Not available	Not available
Cd74	0.577212	0.01278	1.4202	0.062795
Slc39a14	1.500958	0.01287	Not available	Not available
Znf513	0.621718	0.013132	Not available	Not available
Nexn	1.999267	0.013144	1.0396	0.971283
Ttll4	1.761313	0.013148	Not available	Not available
Lrp8	2.485054	0.013327	Not available	Not available
Spsb1	0.392999	0.0137	Not available	Not available
LOC108348617	1.810439	0.013801	Not available	Not available
Prr14l	0.66184	0.013946	Not available	Not available
Adgre5	0.505871	0.014128	Not available	Not available
H2aj	0.310824	0.01429	Not available	Not available
ENSRNOG00000062700	0.543736	0.014767	Not available	Not available
Fbxw2	0.450772	0.014831	Not available	Not available
Fzd7	1.825352	0.014954	Not available	Not available
Apoh	2.047057	0.015597	1.0011	0.948529
Bcl7a	1.760683	0.015938	Not available	Not available

Sdc2	1.605643	0.01603	Not available	Not available
Gpnmb	1.717422	0.016161	1.4751	0.689842
Zscan21	0.283731	0.01621	Not available	Not available
Tent5d	1.847528	0.016287	Not available	Not available
Clic3	0.651151	0.016441	0.9622	0.832608
C4	0.538397	0.01664	Not available	Not available
Nfasc	2.538202	0.017643	Not available	Not available
Hddc3	2.254263	0.017979	0.7542	0.463708
Nap1l1	1.51436	0.018828	1.5177	0.02938
Ocm	2.01126	0.019039	Not available	Not available
Dmwd	0.544186	0.019064	Not available	Not available
Ssna1	1.647839	0.019434	Not available	Not available
Plekhh1	1.813702	0.019496	Not available	Not available
Dglucy	0.47062	0.019885	0.6637	0.022595
Taf1a	0.40294	0.020916	Not available	Not available
Srek1ip1	0.58318	0.021124	Not available	Not available
Mboat7	1.578461	0.021271	1.1335	0.457874
Il1r1	1.543151	0.021456	Not available	Not available
Ap3m1	1.60078	0.021924	0.9972	0.964643
Otud3	0.591	0.021944	Not available	Not available
Cyp4v2	1.874654	0.022257	Not available	Not available
Pds5b	3.95395	0.022331	0.7198	0.207102
Atp2c1	1.822013	0.022703	Not available	Not available
Etl4	0.655033	0.022811	1.0932	0.528337
Zfp61	0.507336	0.022927	Not available	Not available
Zfpm1	0.483368	0.023302	Not available	Not available
Tceanc	0.620691	0.023512	Not available	Not available
Tnfrsf12a	1.752949	0.023771	Not available	Not available
Dync2i2	2.146837	0.023929	Not available	Not available
H3-3b	0.583068	0.024205	Not available	Not available
--	0.520307	0.024932	Not available	Not available
LOC500028	0.566319	0.025023	Not available	Not available
Krcc1	0.357127	0.025731	Not available	Not available
Fgf13	0.501334	0.026239	Not available	Not available
Rragd	0.552125	0.026328	Not available	Not available
Jagn1	1.734187	0.026548	1.0538	0.289009
Thra	0.577254	0.028103	Not available	Not available
Traf3	0.635999	0.02895	Not available	Not available
Krt5	0.224775	0.029011	Not available	Not available
Olfm2	0.486017	0.029242	Not available	Not available
Zcchc9	1.51603	0.029335	Not available	Not available
Rhpn1	0.366966	0.029418	Not available	Not available
Rdh10	0.558957	0.029501	Not available	Not available
Ctso	0.494124	0.02976	Not available	Not available

Tagln3	1.780599	0.029883	Not available	Not available
Tirap	1.87113	0.030335	Not available	Not available
Pdlim2	0.621833	0.030699	1.588	0.012068
Zbtb2	0.516893	0.030716	Not available	Not available
Ifi47	1.761151	0.030772	1.2171	0.352721
Tpp2	1.589	0.030954	1.0256	0.679462
Mageh1	0.50291	0.031042	Not available	Not available
Znf260	2.899252	0.031821	Not available	Not available
Ubald1	0.588212	0.031859	Not available	Not available
Rbms3	0.66115	0.032284	Not available	Not available
AC109542.1	1.51214	0.032301	Not available	Not available
Dedd	0.425766	0.033118	Not available	Not available
Mcoln1	1.610278	0.034058	Not available	Not available
Ntng2	1.703617	0.034764	Not available	Not available
Mcrip2	0.442592	0.034796	Not available	Not available
Efnb1	0.42047	0.034861	1.0342	0.729149
Cdc14a	2.245837	0.03562	Not available	Not available
Dusp23	0.600333	0.035736	Not available	Not available
Tpgs2	2.289164	0.036118	Not available	Not available
Ppcdc	0.579736	0.036476	Not available	Not available
Hnrnpa2b1	0.106097	0.036514	0.955	0.434065
Per1	2.495888	0.036566	Not available	Not available
Rhbdl3	0.509439	0.036934	Not available	Not available
Mx1	0.517885	0.037496	Not available	Not available
Cbx7	0.521296	0.037649	Not available	Not available
Haghl	0.647034	0.038035	Not available	Not available
Ifngr1	1.674358	0.038472	Not available	Not available
Dcun1d2	1.501715	0.038706	Not available	Not available
Retsat	1.624961	0.038877	0.6288	0.029441
Tprn	0.596674	0.039505	Not available	Not available
Jun	0.544125	0.039783	Not available	Not available
Tead3	0.568361	0.040098	Not available	Not available
Ifrd1	1.785552	0.040477	Not available	Not available
Ttc34	1.847643	0.041423	Not available	Not available
Vtcn1	0.572381	0.041481	Not available	Not available
Sft2d3	0.518367	0.041559	Not available	Not available
Daxx	2.15975	0.041712	Not available	Not available
Phactr1	0.485974	0.043296	Not available	Not available
Eml3	0.668544	0.043361	1.4761	0.083015
Hps4	0.291531	0.043539	Not available	Not available
Kctd7	1.602769	0.044138	Not available	Not available
Usp2	1.745822	0.044445	Not available	Not available
Cttnbp2	0.515152	0.044469	Not available	Not available
Tlr4	0.662103	0.044906	Not available	Not available

Macrod2	0.465696	0.045012	Not available	Not available
Slc29a3	0.653259	0.04509	0.7544	0.084562
Ttc7b	1.674227	0.045278	Not available	Not available
St7	2.529013	0.045541	Not available	Not available
Il13ra1	2.086184	0.045991	Not available	Not available
Slc25a36l1	1.617023	0.046354	Not available	Not available
Agtpbp1	0.607488	0.046471	Not available	Not available
Gspt2	0.612975	0.046669	Not available	Not available
Cep350	0.637756	0.047179	Not available	Not available
Rbmx	2.423602	0.04718	1.0558	0.302096
Stkld1	1.93236	0.048299	Not available	Not available
Otulinl	1.881729	0.04841	Not available	Not available
Lrp12	1.53269	0.048594	Not available	Not available
