# Supplementary figures and images for: The Altered Proteomic Landscape in Renal Tubular Epithelial Cells under High Oxalate Stimulation
Source: Biology (Basel). 2024 Oct 11;13(10):814. doi: 10.3390/biology13100814 (PMC11505525; doi:10.3390/biology13100814)

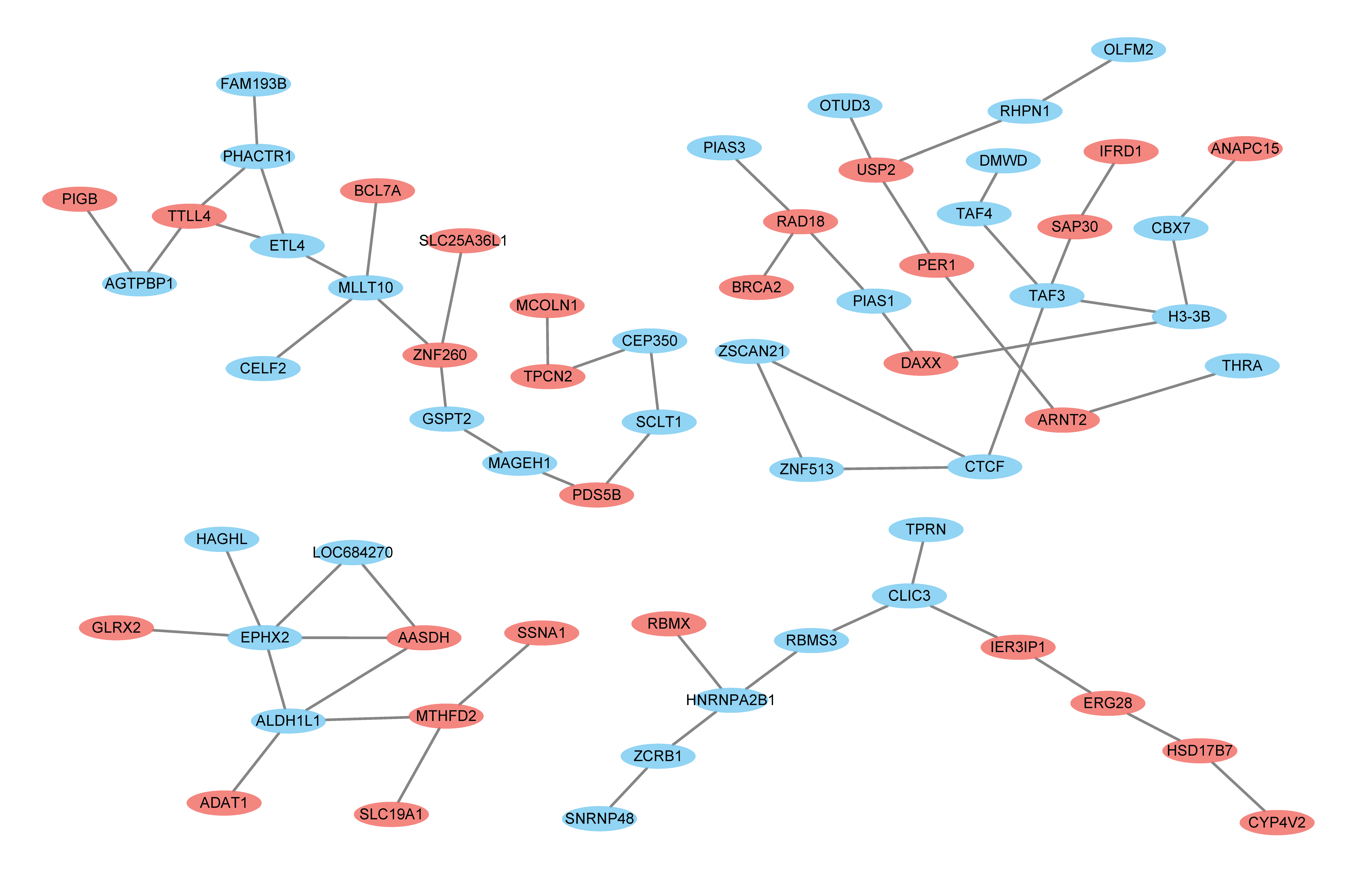

Supplement: Supplementary file 1 [file biology-13-00814-s001.zip › Figure S2.tif]
